# Economic and health burden of severe respiratory syncytial virus infection among hospitalised children at three tertiary hospitals in Argentina, Brazil, and Colombia: a two-year prospective multicentre cohort study

**DOI:** 10.1016/j.lana.2026.101601

**Published:** 2026-08-21

**Authors:** Gonzalo G. Guiñazú, Julia Dvorkin, Pilar Sorgente, Pablo Pakciarz, María Pico, Maria Sol Crespi, Ranniery Acuña, Tamara Schvarstein, Luiz Vicente Ribeiro Ferreira da Silva Filho, Alejandro Colmenares, María de la Paz Restrepo, Cristian Wood, Rocío Lopez Castelo, Talia Soria, Denise Swei Lo, Candela Etchegaray, Clint Pecenka, Gonzalo Perez Marc, Pablo M. Irusta, Romina Libster, Mauricio T. Caballero

**Affiliations:** a Centro Infant de Medicina Traslacional (CIMeT), Universidad Nacional de San Martín, Buenos Aires, Argentina; b Hospital Militar Central, Bogotá, Colombia; c Hospital Militar Central, Buenos Aires, Argentina; d Instituto da Criança e do Adolescente, Hospital das Clínicas, University of São Paulo Medical School, Brazil; e Hospital Universitário da USP (University of São Paulo), São Paulo, Brazil; f Center for Vaccine Innovation and Access, PATH, Seattle, WA, USA; g Department of Human Science, School of Health, Georgetown University, Georgetown, Washington DC, USA; h Fundación INFANT, Buenos Aires, Argentina

**Keywords:** Respiratory syncytial virus (RSV), Lower respiratory tract infection (LRTI), Cost of illness, Disability-adjusted life years (DALYs), Latin America

## Abstract

**Background:**

Respiratory syncytial virus (RSV) is a leading cause of severe lower respiratory tract infection (LRTI) in young children, yet prospective data on its societal and health burden in Latin America remain scarce. We estimated the direct medical, direct non-medical, and indirect costs and the health burden (disability-adjusted life years, DALYs) of severe RSV-associated LRTI, compared with RSV-negative children hospitalised with severe LRTI, in three Latin American countries.

**Methods:**

We conducted a prospective multicentre cohort study between May 2023 and May 2025 among children aged 1–59 months hospitalised with severe LRTI in three tertiary hospitals in Argentina, Brazil, and Colombia. RSV was confirmed by Reverse Transcription Polymerase Chain Reaction (RT-PCR). Individual-level data on healthcare utilisation, household out-of-pocket expenditures, and caregiver productivity losses were collected. Costs were estimated by bottom-up micro-costing and expressed in June 2025 purchasing power parity–adjusted US dollars (USD PPP). DALYs per episode were summarised by country, RSV status, and level of care.

**Findings:**

Among 747 children, 368 (49%) were RSV-positive. Intensive care unit (ICU) admission was the main driver of both costs and DALYs: mean total societal costs per episode were two-to six fold higher for ICU than forward admissions (in Argentina, USD PPP 22,099 vs 3479 among RSV-positive children), and median DALYs were three-to five fold higher (0.0058 vs 0.0011). Costs varied widely across sites but differed minimally by RSV status; differences between RSV-positive and RSV-negative episodes were small and non-significant in all three sites (all 95% confidence intervals [CIs] included zero).

**Interpretation:**

Severe RSV disease imposes a substantial economic and health burden across the three tertiary hospitals studied in Argentina, Brazil, and Colombia. ICU admission, rather than RSV status, is the main determinant of per-episode costs and DALYs, providing evidence to inform policy on RSV prevention.

**Funding:**

Merck Investigator Studies Program (MISP).

## Resumo

**Contexto:**

O vírus sincicial respiratório (VSR) é uma das principais causas de infecção respiratória aguda grave do trato inferior (IRAG) em crianças pequenas; no entanto, os dados prospectivos sobre sua carga social e de saúde na América Latina permanecem escassos. Estimamos os custos médicos diretos, não médicos diretos e indiretos, bem como a carga de saúde (anos de vida ajustados por incapacidade, DALYs) da IRAG grave associada ao VSR, em comparação com crianças VSR-negativas hospitalizadas por IRAG grave, em três países da América Latina.

**Métodos:**

Conduzimos um estudo prospectivo multicêntrico de coorte entre maio de 2023 e maio de 2025 em crianças de 1 a 59 meses hospitalizadas por IRAG grave em três hospitais terciários da Argentina, do Brasil e da Colômbia. A infecção por VSR foi confirmada por reação em cadeia da polimerase com transcrição reversa (RT-PCR). Foram coletados dados individuais sobre utilização de serviços de saúde, gastos diretos das famílias e perdas de produtividade dos cuidadores. Os custos foram estimados por meio de microcusteio ascendente (bottom-up) e expressos em dólares norte-americanos ajustados pela paridade do poder de compra de junho de 2025 (USD PPC). Os DALYs por episódio foram resumidos por local de estudo, status de VSR e nível de cuidado.

**Achados:**

Entre 747 crianças, 368 (49%) foram VSR-positivas. A internação em unidade de terapia intensiva (UTI) foi o principal determinante tanto dos custos quanto dos DALYs: os custos sociais totais médios por episódio foram de duas a seis vezes superiores nas internações em UTI do que nas internações em enfermaria (na Argentina, 22.099 frente a 3.479 USD PPC em crianças VSR-positivas), e os DALYs medianos foram de três a cinco vezes superiores (0.0058 frente a 0.0011). Os custos variaram amplamente entre os locais, mas diferiram minimamente conforme o status de VSR; as diferenças entre episódios VSR-positivos e VSR-negativos foram pequenas e não significativas nos três locais (todos os IC de 95% incluíram zero).

**Interpretação:**

A doença grave por VSR impôs uma carga econômica e de saúde substancial nos três hospitais terciários estudados na Argentina, no Brasil e na Colômbia. A internação em UTI, mais do que o status de VSR, é o principal determinante dos custos e dos DALYs por episódio, fornecendo evidências para orientar as políticas de prevenção do VSR.

## Abstract (es)

**Antecedentes:**

El virus sincicial respiratorio (VSR) es una de las principales causas de infección respiratoria aguda baja (IRAB) grave en niños pequeños; sin embargo, los datos prospectivos sobre su carga social y sanitaria en América Latina siguen siendo escasos. Estimamos los costos médicos directos, no médicos directos e indirectos, así como la carga sanitaria (años de vida ajustados por discapacidad, AVAD) de la IRAB grave asociada al VSR, en comparación con niños VSR-negativos hospitalizados por IRAB grave, en tres países de América Latina.

**Métodos:**

Realizamos un estudio prospectivo multicéntrico de cohorte entre mayo de 2023 y mayo de 2025 en niños de 1 a 59 meses hospitalizados por IRAB grave en tres hospitales de tercer nivel de Argentina, Brasil y Colombia. La infección por VSR se confirmó mediante reacción en cadena de la polimerasa con transcripción inversa (RT-PCR). Se recolectaron datos individuales sobre utilización de servicios de salud, gastos de bolsillo de los hogares y pérdidas de productividad de los cuidadores. Los costos se estimaron mediante un enfoque de microcosteo ascendente (bottom-up) y se expresaron en dólares estadounidenses ajustados por paridad de poder adquisitivo de junio de 2025 (USD PPA). Los AVAD por episodio se resumieron por sitio, estado de VSR y nivel de atención.

**Hallazgos:**

Entre 747 niños, 368 (49%) fueron VSR-positivos. La internación en unidad de cuidados intensivos (UCI) fue el principal determinante tanto de los costos como de los AVAD: los costos sociales totales medios por episodio fueron de dos a seis veces mayores en las internaciones en UCI que en sala general (en Argentina, 22.099 frente a 3.479 USD PPA en niños VSR-positivos), y los AVAD medianos fueron de tres a cinco veces mayores (0,0058 frente a 0,0011). Los costos variaron ampliamente entre los sitios, pero difirieron mínimamente según el estado de VSR; las diferencias entre episodios VSR-positivos y VSR-negativos fueron pequeñas y no significativas en los tres sitios (todos los IC del 95% incluyeron el cero).

**Interpretación:**

La enfermedad grave por VSR impuso una carga económica y sanitaria sustancial en los tres hospitales de tercer nivel estudiados en Argentina, Brasil y Colombia. La internación en UCI, más que el estado de VSR, es el principal determinante de los costos y los AVAD por episodio, lo que aporta evidencia para orientar las políticas de prevención del VSR.

**Research in context**

**Evidence before this study**

Respiratory syncytial virus (RSV) is a leading cause of lower respiratory tract infection and hospitalisation among young children globally, with high burden in low- and middle-income countries. Previous studies have documented the clinical and economic impact of RSV; however, after searching PubMed/MEDLINE and LILACS (January 2012–March 2026) using terms combining “RSV”, “economic burden”, “cost”, and "Latin America” or individual country names, we found that evidence from Latin America remains limited, fragmented, and methodologically heterogeneous. Most available studies are retrospective, single-country analyses, and frequently restricted to direct medical costs from a payer perspective. Few studies have prospectively collected individual-level data, and even fewer have incorporated direct non-medical costs, caregiver productivity losses, or standardized measures of health burden such as disability adjusted life years (DALYs). As a result, cross-country comparisons and comprehensive assessments of the societal burden of RSV in the region remain scarce.

**Added value of this study**

This study addresses a critical evidence gap in Latin America, with prospective, patient-level data collected in parallel at hospitals in three countries. It extends existing knowledge in three ways: by moving beyond the payer perspective—predominant in prior regional studies—to a full societal perspective that incorporates household and indirect costs; by adding a standardized measure of health loss (DALYs) alongside costs, rarely available in hospital-based RSV studies in the region; and by showing that burden patterns were consistent across three tertiary hospitals embedded in structurally different health systems, suggesting that the observed drivers of cost are not specific to a single setting. Together, these contributions provide a comparable evidence base that was previously unavailable for the region.

**Implications of all the available evidence**

These findings suggest that the burden of severe RSV disease among children hospitalised at tertiary centres in Latin America is substantial and largely driven by disease severity and intensity of care. The consistency of cost gradients across the three participating hospitals underscores the importance of preventing severe disease requiring hospitalisation and intensive care. By providing harmonized, prospective estimates from three tertiary hospitals in three countries from a societal perspective, this study offers critical inputs for economic evaluations and budget impact analyses of emerging RSV preventive strategies, including long-acting monoclonal antibodies and maternal vaccination. These data can support evidence-based policy decisions on prioritisation and implementation of RSV prevention programmes in the region.

## Introduction

Respiratory syncytial virus (RSV) is the leading viral cause of lower respiratory tract infections (LRTI) in infants and young children worldwide.^1^ Virtually all children are infected in their second year of life, with reinfections occurring throughout early childhood^2^ and recent global estimates attribute more than 33 million RSV-associated LRTI episodes, about 3.6 million hospitalisations, and nearly 1 in 50 deaths among children aged 0–59 months of age to RSV, underscoring a substantial and persistent global burden concentrated in low- and middle-income countries (LMICs).^3^, ^4^, ^5^ In response to this considerable disease burden, four licenced RSV preventive interventions are currently available for young infants. Palivizumab remains indicated for high-risk preterm infants but requires five monthly doses, with limited applicability for most LMICs due to its high cost.^6^ More recently, two long-acting monoclonal antibodies show strong efficacy against RSV-associated LRTI: nirsevimab, and clesrovimab, both of which provide season-long protection with a single dose.^7^, ^8^, ^9^ Complementing these alternatives, the maternal RSV prefusion F (RSVpreF) vaccine confers infants’ protection through transplacental antibodies during the first six months of life.^10^^,^^11^ However, these strategies currently have limited availability in many LMIC settings.

RSV also imposes a substantial economic strain on health systems, households, and broader economies worldwide.^12^^,^^13^ The global annual cost of RSV has been estimated at roughly €4.82 billion, with nearly 65% occurring in LMICs.^12^ Hospital care usually requires oxygen therapy, laboratory and imaging tests, and prolonged inpatient stays, while caregiver productivity losses further increase the total burden.^14^, ^15^, ^16^, ^17^, ^18^ However, evidence from Latin America remains limited and methodologically heterogeneous, often restricted to single-country estimates, which hampers regional comparisons.^19^, ^20^, ^21^

Comprehensively characterising the epidemiological and economic impact of RSV is therefore essential for informing health policy and anticipating the potential value of emerging preventive technologies. Yet important methodological gaps persist: most available studies are retrospective, payer-based and rarely include non-medical or indirect costs.^19^ As Latin American countries evaluate adoption pathways and financing strategies, decision-makers increasingly require harmonised, prospectively collected evidence that reflects the full economic and health burden of RSV and allows meaningful comparisons. To address these gaps, we conducted a prospective, multi-country study in hospitalised children under five years across tertiary hospitals in Argentina, Brazil, and Colombia. Our primary objective was to quantify the economic (direct medical, direct non-medical, and indirect) and health (DALY) burden of severe RSV-associated LRTI, and to compare it with that of RSV-negative children hospitalised with severe LRTI of comparable clinical severity.

## Methods

### Study design and setting

We conducted a prospective, multicentre cohort study from May 7, 2023 to May 7, 2025, across three tertiary referral hospitals in major urban cities of Latin America: Hospital Militar Central Cirujano Mayor Dr. Cosme Argerich (Buenos Aires, Argentina), Hospital Universitário da USP (University of São Paulo, Brazil), and Hospital Militar Central (Bogotá, Colombia). Standardised procedures and instruments — including harmonised case report forms, structured caregiver interview questionnaires, and standardised case definitions (detailed in Data Collection and Data Management) — were applied across all sites to ensure comparability of clinical, epidemiological, and economic data while allowing sufficient flexibility to capture local differences in healthcare delivery and cost structures This study is reported in accordance with the Consolidated Health Economic Evaluation Reporting Standards 2022 (CHEERS 2022)^22^ and the Strengthening the Reporting of Observational Studies in Epidemiology (STROBE)^23^ guidelines (checklists provided as separate files).

### Study population

Eligible participants were post-neonatal infants and children aged >28 days and <5 years admitted with severe LRTI, defined as the presence of at least one manifestation of lower respiratory tract infection sign (cough, nasal flaring, indrawing of the lower chest wall, subcostal retractions, stridor, rales, rhonchi, wheezing, crackles or crepitations, or observed apnoea) plus at least one of hypoxaemia (peripheral oxygen saturation of <95% at room air for Argentina and Brazil and <90% for Colombia) or tachypnoea (≥70 breaths per minute from 0 to 59 days of age, and ≥ 60 breaths per minute at 60 days of age or older).^24^, ^25^, ^26^

Children were excluded if they resided outside the metropolitan catchment area of the participating hospitals, were readmitted for LRTI within one month of a prior discharge, or if their parents or guardians declined to provide informed consent. Consecutive enrolment of all eligible patients was performed at each site to minimise selection bias.

### Data collection and data management

After parental consent, trained investigators collected demographic, epidemiological, clinical, and economic information through medical record review and structured caregiver interviews (questionnaire provided in Supplementary Material). Data on sociodemographic and environmental characteristics were obtained by investigators through review of medical records using the study's standardised data collection form. Sex was recorded as biological sex (female/male) as documented in the medical record. Data on gender identity were not collected, as the study relied exclusively on clinical information abstracted from medical records. Data on race and ethnicity were not collected, as these variables are not routinely recorded in the medical records that served as the data source for this study. Clinical information encompassed perinatal and medical history, comorbidities, presenting symptoms, disease course, laboratory and imaging findings, requirements for respiratory support, admission to the paediatric intensive care unit (ICU), and outcome classification at discharge. Missing data were infrequent across the analysed variables, as information was abstracted from standardised case report forms and complete medical records. No variable had more than 1% of missing observations overall, and the highest proportion at any single site was 1.3%; the most frequent was non-completion of the caregiver interview (7/747 [0.9%]), which precluded estimation of household out-of-pocket and indirect costs for those participants. The number and proportion of missing observations for each variable are reported in Supplementary Table S1. Complete-case analysis was applied and no imputation was performed; the effective denominator for each comparison is therefore the number of participants with complete data for the corresponding variable. Data on healthcare utilisation and costs were obtained from hospital administrative and medical records. Cost components included personnel, overhead, diagnostics, medications, nutrition, daily bed charges, and supportive therapies. Indicators of disease severity, such as duration of supplemental oxygen, ICU admission, number of days on mechanical ventilation, and length of hospital stay (LOS) were also captured. All the data enabled the calculation of direct medical costs from the provider perspective. Out-of-pocket and indirect costs were assessed using structured research questionnaires administered to caregivers during hospitalisation. Items included transport to and from the hospital, meals, accommodation, hired caregivers, and opportunity costs derived from income lost due to caregiving responsibilities. Household expenditures were combined with direct medical costs to generate estimates from the societal perspective. For each patient, detailed resource utilisation was recorded at the individual level, including days in paediatric ward and ICU, use and duration of respiratory support modalities (high-flow nasal cannula, non-invasive ventilation, invasive mechanical ventilation), imaging studies, laboratory tests, medications, and nutritional support. For each resource, both a binary indicator of use (yes/no) and a quantitative measure of intensity (e.g., days, doses, or tests) were captured, allowing estimation of both utilisation rates and per-patient consumption across disease severity strata and countries.

All clinical and economic data were entered into REDCap™, designed for the study.^27^ Each participant was assigned a unique alphanumeric identifier, which was consistently used across case report forms and laboratory samples. Variable dictionaries and coding schemes were harmonised across sites. All research forms were available both in Spanish and Portuguese. Data quality was ensured through continuous monitoring procedures, including systematic data-cleaning checks, cross-form consistency reviews, and routine curation throughout the study period. In addition, site visits and independent audit inspections were conducted at all three participating centres to verify protocol adherence, source-document accuracy, and completeness of case records.

### Ethical considerations

The study posed minimal risk and followed routine clinical care, with ethics approval obtained from all Institutional Review Boards of the participating centres (Hospital Militar Central Buenos Aires approval ID PRIISA.BA 6779; Hospital Universitario da USP approval ID 5.600.127; Hospital Militar Central Bogotá approval ID 2022062). Written informed consent was obtained from parents or guardians. All procedures adhered to international and local ethical guidelines.^28^, ^29^, ^30^ Details on biological sample collection and viral diagnostics are provided in the Supplementary Methods.

### Outcomes

The primary outcomes were the economic burden (direct medical, direct non-medical, and indirect costs) and health burden (DALYs) per hospitalised episode of severe LRTI. RSV status, confirmed uniformly by centralised RT-PCR (Reverse Transcription Polymerase Chain Reaction), defined the two comparison groups (RSV-positive and RSV-negative). No point-of-care or rapid antigen testing was used for classification. Secondary clinical outcomes included ICU admission, invasive mechanical ventilation, length of hospital stay, and in-hospital mortality. Economic outcomes were assessed from a societal perspective and included direct medical costs (provider perspective), direct non-medical costs (household perspective), and indirect costs due to caregiver productivity losses. Health care utilisation outcomes comprised both utilisation rates and mean quantities of use per patient, forming the basis for all cost calculations.

### Health burden (DALYs)

We quantified the health burden attributable to RSV using disability-adjusted life years (DALYs), following standard Global Burden of Disease (GBD) methodology.^31^, ^32^, ^33^ DALYs were calculated as the sum of years of life lost (YLL) due to premature mortality and years lived with disability (YLD).

YLLs were estimated for in-hospital deaths using remaining life expectancy from the GBD standard life table matched to age in months. YLDs were calculated by multiplying episode duration (length of stay) by the corresponding GBD disability weights for severe and life-threatening LRTI, defined by ward or ICU admission, respectively. Because DALYs showed strong right-skewness driven by rare fatal events, results were primarily summarised using medians and interquartile ranges.

### Sample size and statistical analysis

The sample size was calculated to estimate mean hospitalisation costs with a relative precision of ±10%, assuming a coefficient of variation of 0.5.^34^ Based on an expected RSV positivity of 50% among hospitalised severe LRTI cases, a minimum of 292 LRTI cases was required. To account for seasonal variability, a 10% margin was added, resulting in a target sample of 321 children.^35^

Costs were estimated at the individual-patient level using a bottom-up micro-costing approach.^36^^,^^37^ Country-specific unit costs were applied to observed quantities of resource use to generate per-patient cost estimates. Direct medical costs included bed-day charges, medications (including antibiotics and bronchodilators), diagnostics (imaging, laboratory tests, and microbiological cultures), and respiratory support (oxygen therapy via nasal cannula, Venturi mask, reservoir mask, or high-flow nasal cannula, and non-invasive or invasive mechanical ventilation). Direct non-medical costs comprised household expenditures and allocated hospital overhead. Indirect costs were estimated as caregiver productivity losses, monetised using country-specific minimum hourly wages.^38^^,^^39^ All costs were expressed in June 2025 purchasing power parity–adjusted US dollars (USD PPP).^40^

Given the substantial structural heterogeneity across the three health systems in prices, financing, and case-mix, all analyses were fully stratified by country, RSV status, and level of care rather than pooled across countries. This approach reflects one of the two standard strategies for multinational cost data and was chosen to preserve country-specific interpretability, which is most relevant for national decision-making, since pooled estimates may be difficult to interpret for any individual country.^41^ Descriptive analyses were performed for all variables. Continuous data were summarised as means (standard deviation, SD) or medians (interquartile ranges, IQR), and categorical variables as frequencies and percentages. Baseline demographic and clinical characteristics were compared between RSV-positive and RSV-negative children within each country using the Wilcoxon rank-sum (Mann–Whitney U) test for continuous variables and the χ^2^ test or Fisher's exact test (for expected cell counts <5) for categorical variables. Mean costs were estimated separately for RSV-positive and RSV-negative hospitalisations. Given the pronounced right-skewness of cost data, both uncertainty and between-group comparisons for costs were derived from the same non-parametric bootstrap procedure (1000 resamples), consistent with recommended methods for analysing skewed cost data^42^: 95% confidence intervals (CIs) were obtained from the bootstrap distribution of mean costs, and differences between RSV-positive and RSV-negative groups were considered statistically significant when the bootstrap 95% confidence interval of the mean difference excluded zero. Key cost drivers, including ICU admission, length of stay, and caregiver absenteeism, were explored descriptively using stratified analyses.^43^^,^^44^ Because analyses were descriptive and estimative in nature, with no single confirmatory primary hypothesis, no adjustment for multiple comparisons was applied. All p-values are two-sided, with statistical significance set at 0.05. Analyses were performed in Stata (Stata Corp LLC, College Station, TX, USA), version 17. As a pre-specified secondary sensitivity analysis, these cost components were also re-expressed using the June 2025 official exchange rate instead of PPP-adjusted values, given Argentina's high-inflation context. Detailed costing and statistical methods are explained in Supplementary Methods.

### Role of the funding source

The funder had no role in study design, data collection, data analysis, data interpretation, or writing of the report.

## Results

### Study population and RSV epidemiology

Between May 7, 2023 and May 7, 2025, 1682 patients hospitalised with severe LRTI were assessed for eligibility. Of these, 802 did not meet the inclusion criteria, and 133 of the 880 eligible patients were excluded (102 declined informed consent; 31 for other reasons), leaving 747 children included in the analysis (Supplementary Figure S1). Enrolment comprised 229 admissions in Argentina, 294 in Brazil, and 224 in Colombia. Overall, 49% (368/747) of hospitalised children tested positive for RSV with substantial variation across hospitals, ranging from 37% in Argentina (85/229) to **56% in Brazil (166/294)** and 52% in Colombia (117/224). Temporal analysis demonstrated marked seasonality in Argentina and Colombia, with well-defined winter peaks, whereas Brazil showed more sustained RSV circulation throughout the year (Supplementary Figure S2).

### Baseline demographic and clinical characteristics

Baseline demographic and clinical characteristics stratified by RSV status and country are shown in Table 1. Age patterns differed across sites. In Argentina, RSV-positive children were significantly younger than RSV-negative children (median 17.0 [8.0–28.0] vs 22.0 [11.0–38.0] months, p = 0.016), whereas in Colombia RSV-positive children were significantly older (18.0 [6.0–34.0] vs 11.0 [4.0–30.0] months, p = 0.039). No significant difference by RSV status was observed in Brazil (5.0 [3.0–10.0] vs 6.5 [4.0–13.0] months, p = 0.23), where enrolled children were younger overall than at the other two sites. Sex distribution and prematurity rates were similar between RSV-positive and RSV-negative children across all sites. Prior LRTI-related hospitalisation did not differ significantly by RSV status at any site, although it tended to be more frequent among RSV-negative children in Brazil (31/166 [19%] vs 35/128 [27%], p = 0.08) and Colombia (37/117 [32%] vs 45/107 [42%], p = 0.11). Mean total length of hospital stay did not differ significantly by RSV status at any site and ranged from approximately 5 to 6 days across sites.

**Table 1:** Demographics of RSV positive and RSV negative in children <5 years old by study site.^a^

	Argentina			Brazil			Colombia		
	RSV + (n = 85)	RSV − (n = 144)	p	RSV + (n = 166)	RSV − (n = 128)	p	RSV + (n = 117)	RSV − (n = 107)	p
Age in months, median (IQR)	17.0 (8.0–28.0)	22.0 (11.0–38.0)	**0.016**	5.0 (3.0–10.0)	6.5 (4.0–13.0)	0.23	18.0 (6.0–34.0)	11.0 (4.0–30.0)	0.039
Sex Female, n (%)	46 (54)	73 (51)	0.63	79 (48)	69 (54)	0.28	51 (44)	48 (45)	0.85
Prematurity, n (%)	19 (22)	34 (24)	0.69	25 (15)	22 (17)	0.89	32 (27)	22 (21)	0.68
Comorbidities, n (%)	25 (29)	55 (38)	0.18	18 (11)	19 (15)	0.63	18 (15)	21 (20)	0.16
Prior LRTI hospitalisation, n (%)	26 (31)	53 (37)	0.34	31 (19)	35 (27)	0.08	37 (32)	45 (42)	0.11
Total length of stay, $\bar{x}$ (±SD)	5.2 (4.0)	5.0 (5.8)	0.71	6.3 (4.1)	5.7 (5.3)	0.32	6.1 (2.8)	5.7 (3.8)	0.45
ICU admission, n (%)	8 (9)	14 (10)	0.94	69 (42)	35 (27)	**0.011**	8 (7)	9 (8)	0.66
MV requirement, n (%)	4 (5)	6 (4)	0.85	15 (9)	5 (4)	0.08	0	0	–

Data are n (%) unless otherwise stated. Age is presented as median (IQR) and total length of stay as mean (SD). ICU, intensive care unit; IQR, interquartile range; LRTI, lower respiratory tract infection; MV, mechanical ventilation; RSV, respiratory syncytial virus; SD, standard deviation.

p values shown in bold indicate statistically significant between-group differences.

a Study sites: Hospital Militar Central Cosme Argerich (Buenos Aires, Argentina); Hospital Universitário da USP (São Paulo, Brazil); Hospital Militar Central (Bogotá, Colombia). Data are not representative at the national level.

### Clinical severity and healthcare utilisation

Markers of clinical severity varied substantially across sites (Table 1). In Brazil, RSV-positive children compared with RSV-negative children experienced higher rates of ICU admission (69/166 [42%] vs 35/128 [27%], p = 0.011). In contrast, ICU admission rates in Argentina (8/85 [9%] vs 14/144 [10%], p = 0.94) and Colombia (8/117 [7%] vs 9/107 [8%], p = 0.66) were lower and did not differ significantly by RSV status. Mechanical ventilation was uncommon overall. In Brazil, mechanical ventilation was required in 15/166 [9%] of RSV-positive children compared with 5/128 [4%] of RSV-negative children, although this difference did not reach statistical significance (p = 0.08). In Argentina, rates were low and similar between groups (4/85 [5%] vs 6/144 [4%], p = 0.85), while no cases of mechanical ventilation were observed in Colombia. Overall, in-hospital mortality was rare, with a single death occurring in an RSV-negative child in Brazil.

Table 2 presents data on healthcare resource utilisation by study site and RSV status, further stratified by level of care (general ward and paediatric intensive care unit). The table summarises the frequency of diagnostic testing, respiratory support modalities, and pharmacologic treatments across settings. Use of advanced respiratory support (high-flow nasal cannula, non-invasive ventilation, and invasive mechanical ventilation) was concentrated among ICU patients and showed marked inter-site variation. In Brazil, high-flow nasal cannula was used in 56/69 [81%] of RSV-positive ICU admissions compared with 23/35 [66%] among RSV-negative ICU patients. Detailed resource utilisation is presented in Supplementary Table S2.

**Table 2:** Resource utilisation during hospitalisation among children younger than five years with LRTI, by study site.^a^

	Argentina						Brazil						Colombia					
	Ward			ICU			Ward			ICU			Ward			ICU		
	RSV (+)	RSV (−)	p value	RSV (+)	RSV (−)	p value	RSV (+)	RSV (−)	p value	RSV (+)	RSV (−)	p value	RSV (+)	RSV (−)	p value	RSV (+)	RSV (−)	p value
Total n (%)	77 (37)	130 (63)		8 (36)	14 (64)		97 (51)	93 (49)		69 (73)	35 (27)		109 (53)	98 (47)		8 (47)	9 (53)	
Any antibiotic n (%)	25/77 (33)	30/130 (23)	0.15	7/8 (88)	10/14 (71)	0.37	20/97 (21)	24/93 (26)	0.41	42/69 (61)	22/35 (63)	0.84	17/109 (16)	24/98 (25)	0.09	4/8 (50)	5/9 (56)	0.80
Any corticosteroid n (%)	24/77 (31)	54/130 (42)	0.18	6/8 (75)	13/14 (93)	0.59	43/97 (44)	44/93 (44)	0.64	26/69 (38)	14/35 (40)	0.74	42/109 (39)	33/98 (34)	0.38	6/8 (75)	6/9 (67)	>0.99
Any bronchodilator n (%)	70/77 (91)	113/130 (87)	0.53	7/8 (88)	14/14 (100)	>0.99	65/97 (67)	66/93 (71)	0.59	39/69 (57)	19/35 (54)	0.85	95/109 (87)	63/98 (64)	**<0.0001**	7/8 (88)	6/9 (67)	0.58
Any analgesic n (%)	9/77 (12)	11/130 (9)	0.49	5/8 (63)	3/14 (21)	0.07	58/97 (60)	51/93 (55)	0.52	58/69 (84)	26/35 (74)	0.26	43/109 (39)	36/98 (37)	0.70	6/8 (75)	4/9 (44)	0.19
Any vasoactive drug n (%)	0	0	–	3/8 (38)	2/14 (14)	0.30	3/97 (3)	3/93 (3)	>0.99	14/69 (20)	6/35 (17)	0.22	1/109 (1)	2/98 (2)	0.57	1/8 (13)	1/9 (11)	>0.99
Any sedation-analgesia n (%)	1/77 (1)	0	0.36	6/8 (75)	7/14 (50)	0.22	1/97 (1)	0	0.50	37/69 (54)	22/35 (63)	0.35	0	0	–	1/8 (13)	1/9 (11)	>0.99
Any diuretic n (%)	0	0	–	4/8 (50)	6/14 (43)	0.74	1/97 (1)	0	0.30	11/69 (16)	4/35 (11)	0.54	1/109 (1)	0	0.32	0	2/9 (22)	0.47
Any imaging n (%)	52/77 (68)	77/130 (59)	0.24	8/8 (100)	13/14 (93)	0.39	65/97 (67)	71/93 (76)	0.16	69/69 (100)	35/35 (100)	>0.99	92/109 (84)	84/98 (86)	0.81	8/8 (100)	9/9 (100)	>0.99
Any laboratory test n (%)	6/77 (8)	20/130 (15)	0.11	7/8 (88)	9/14 (64)	0.24	96/97 (99)	91/93 (98)	0.54	69/69 (100)	35/35 (100)	>0.99	105/109 (96)	98/98 (100)	0.06	8/8 (100)	9/9 (100)	>0.99
Any oxygen requirement n (%)	76/77 (99)	126/130 (97)	>0.99	8/8 (100)	14/14 (100)	–	91/97 (94)	83/93 (89)	0.40	69/69 (100.0)	34/35 (97)	0.34	107/109 (98)	96/98 (98)	>0.99	8/8 (100)	9/9 (100)	–

ICU, intensive care unit; LRTI, lower respiratory tract infection; RSV, respiratory syncytial virus.

p values shown in bold indicate statistically significant between-group differences.

a Study sites: Argentina, Hospital Militar Central “Cirujano Mayor Dr. Cosme Argerich” (Buenos Aires); Brazil, Hospital Universitário da USP (São Paulo); Colombia, Hospital Militar Central (Bogotá). Data are not representative at the national level—indicates that the comparison was not calculable.

### Direct medical costs per hospitalisation episode

Mean direct medical costs per hospitalisation episode differed markedly across sites and by disease severity (Table 3).

**Table 3:** Societal cost (in 2025 PPP USD) per LRTI episode for RSV-positive and RSV-negative children <5 years by study site.^a^

	Argentina				Brazil				Colombia			
	RSV (+)		RSV (−)		RSV (+)		RSV (−)		RSV (+)		RSV (−)	
	Ward (n = 77)	ICU (n = 8)	Ward (n = 130)	ICU (n = 14)	Ward (n = 97)	ICU (n = 69)	Ward (n = 93)	ICU (n = 35)	Ward (n = 109)	ICU (n = 8)	Ward (n = 98)	ICU (n = 9)
Total costs	3479.22 (3011.67–3946.77)	22,099.02 (11,537.74–32,660.30)	3249.91 (2705.51–3794.31)	12,252.92 (7997.40–16,508.43)	959.15 (853.55–1064.74)	3769.76 (3110.53–4428.99)	885.20 (796.46–973.94)	3639.20 (2398.54–4879.87)	2593.32 (2381.74–2804.91)	6002.29 (3410.34–8594.23)	2346.77 (2132.12–2561.42)	7933.06 (2872.16–12,993.95)
Direct costs	3394.33 (2947.05–3841.62)	22,015.77 (11,274.79–32,756.76)	3177.41 (2643.88–3710.93)	12,191.57 (7995.71–16,387.42)	900.52 (794.46–1006.57)	3682.11 (3023.25–4340.97)	836.02 (754.14–917.89)	3539.64 (2341.52–4737.77)	2393.35 (2195.22–2591.49)	5576.13 (3271.05–7881.21)	2146.52 (1943.11–2349.93)	7537.95 (2432.48–12,643.42)
Direct medical costs	3188.98 (2784.60–3593.36)	21,457.68 (10,946.91–31,968.45)	3018.56 (2504.77–3532.35)	11,771.49 (7721.52–15,821.47)	675.52 (592.81–758.22)	3080.54 (2489.38–3671.69)	623.83 (561.27–686.38)	2935.23 (1853.89–4016.57)	1974.17 (1802.94–2145.39)	4912.98 (2800.98–7024.98)	1770.25 (1598.35–1942.14)	6665.08 (1827.34–11,502.83)
Bed-day cost	2490.93 (2148.97–2836.50)	11,260.05 (6867.39–15,405.23)	2366.61 (1963.65–2865.52)	6717.14 (4313.11–9262.58)	539.71 (470.42–612.04)	2276.18 (1920.74–2645.29)	491.93 (443.75–538.84)	2075.22 (1509.25–2762.46)	1658.29 (1507.41–1813.17)	4101.07 (2460.64–5905.53)	1479.10 (1330.60–1640.97)	4957.73 (2478.87–9186.39)
Total Medications costs	142.62 (114.93–173.82)	5461.62 (915.93–11,319.82)	150.00 (124.90–177.37)	1804.83 (962.22–2948.64)	28.2 (21.00–37.00)	150.36 (118.18–183.87)	31.64 (23.97–39.92)	166.50 (122.00–223.25)	39.26 (26.28–54.56)	135.43 (40.33–255.57)	24.51 (17.37–32.33)	168.47 (42.23–337.93)
Total antibiotics costs	14.49 (7.19–22.93)	2125.65^b^ (149.34–5013.06)	14.65 (6.74–23.86)	719.62^a^ (167.77–1464.56)	10.70 (5.20–17.98)	50.99 (37.42–66.21)	16.24 (9.79–23.50)	71.40 (43.18–108.14)	1.87 (1.04–2.77)	19.73 (−)	5.46 (3.23–8.54)	46.19 (3.95–102.77)
Total bronchodilators	74.63 (62.15–85.97)	270.61 (150.46–406.23)	81.86 (66.79–100.36)	261.76 (169.38–357.39)	10.54 (8.02–13.15)	23.79 (16.37–31.20)	10.97 (8.82–13.55)	11.65 (6.04–17.82)	32.22 (20.52–46.59)	189.16 (37.15–341.16)	14.71 (8.52–21.16)	104.07 (3.28–277.25)
Total imaging	26.61 (20.44–34.32)	123.49 (69.66–181.15)	25.55 (20.07–32.40)	68.44 (44.33–94.53)	5.76 (3.42–9.81)	54.74 (43.94–65.10)	4.21 (3.49–4.96)	29.76 (15.14–49.80)	114.68 (91.73–140.16)	222.78 (89.62–355.93)	117.25 (92.32–145.98)	535.64 (267.27–860.90)
Total laboratory tests	0.94 (0.19–2.00)	28.81 (14.01–46.25)	1.37 (0.63–2.41)	22.24 (7.54–42.68)	2.18 (1.03–3.63)	6.70 (1.67–11.72)	0.85 (0.45–1.31)	22.30 (14.75–32.22)	19.77 (12.69–27.27)	71.30 (34.44–108.16)	18.85 (12.85–26.30)	430.91 (137.38–831.06)
Total Microbiological Culture	16.02 (7.46–26.26)	153.93 (66.69–290.41)	7.70 (3.09–13.13)	83.86 (41.33–133.71)	1.04 (0.51–1.70)	8.48 (6.29–10.77)	0.74 (0.37–1.17)	7.96 (5.42–10.98)	2.64 (−)	25.48 (−)	3.35 (−)	53.01 (−)
Total oxygen requirement	218.81 (152.9–322.6)	4132.81 (2495.8–6063.7)	168.13 (140.3–199.9)	2672.01 (1792.4–3578.5)	60.62 (50.1–71.6)	563.48 (340.66–786.29)	56.07 (46.3–65.5)	588.06 (167.9–1195.7)	47.10 (41.0–54.1)	235.35 (163.12–307.57)	36.95 (32.7–41.6)	371.91 (145.3–763.9)
Direct non-medical costs	205.35 (141.04–269.65)	558.09 (321.41–794.76)	158.84 (131.95–185.73)	420.07 (249.56–590.58)	225.00 (200.36–249.63)	601.57 (523.83–679.30)	212.19 (190.72–233.65)	604.40 (460.30–748.51)	419.18 (382.22–456.15)	663.15 (414.39–911.91)	376.27 (336.34–416.20)	872.86 (516.00–1229.72)
Operational Overhead^c^	91.75 (79.48–104.03)	487.64 (298.64–676.40)	87.17 (69.96–104.40)	374.92 (247.77–502.07)	168.31 (147.11–189.41)	488.05 (218.56–343.87)	153.42 (137.78–168.94)	526.24 (394.97–657.50)	221.56 (201.81–241.32)	442.37 (323.62–561.12)	197.62 (177.45–217.79)	531.50 (264.90–798.08)
Transportation	54.20 (25.01–83.39)	60.59 (−)	34.84 (23.70–45.98)	34.22 (−)	47.30 (39.40–55.21)	91.12 (64.20–118.05)	46.49 (62.92–82.94)	66.42 (41.86–104.01)	120.82 (102.70–138.93)	142.64 (52.89–232.39)	114.38 (96.29–132.47)	196.63 (134.39–258.87)
Accommodation	0	0	0	0	0	0	0	0	0	0	0.68 (−)	78.02 (−)
Paid Caregiver	29.20 (0–75.00)	0	12.86 (4.98–20.74)	9.90 (−)	5.44 (1.16–9.71)	19.42 (7.49–31.34)	8.85 (4.04–13.65)	8.78 (1.39–16.16)	45.85 (28.53–63.15)	0	37.01 (21.11–52.90)	42.13 (−)
Meals	30.18 (19.47–40.90)	9.86 (−)	23.96 (17.16–30.75)	1.03 (−)	3.99 (2.56–5.43)	2.97 (1.63–4.30)	3.48 (2.27–4.70)	2.98 (1.77–4.18)	30.95 (22.34–39.56)	0	26.57 (21.49–31.64)	24.57 (14.59–34.56)
Total indirect costs (productivity losses)	84.89 (61.07–108.70)	83.25 (−)	72.51 (51.45–93.56)	61.35 (−)	58.63 (43.45–73.81)	87.65 (−)	49.19 (33.98–64.39)	99.56 (57.60–141.52)	199.97 (158.93–241.01)	426.16 (267,03–588,84)	200.25 (158.43–242.06)	395.11 (151.88–638.34)

a Given the highly right-skewed distribution of cost data and the presence of a large proportion of zero values, percentile-based bootstrap confidence intervals were estimated for mean costs. This approach avoids reliance on normality assumptions and prevents the generation of implausible negative confidence limits, providing more robust and interpretable uncertainty estimates for average per-patient costs. Bootstrap confidence intervals were not estimated for country–severity strata with fewer than five observations with non-zero costs, as resampling in very small effective samples leads to unstable and non-informative interval estimates.

b In Argentina ICU patients, antibiotic costs were highly right-skewed; the median [IQR] cost was USD 244 [109–2330], substantially lower than the mean. Among non-ICU Argentine patients, median [IQR] antibiotic costs were USD 226 [0–516] in RSV-positive cases and USD 0 [0–8] in RSV-negative cases, reflecting infrequent use with occasional high-cost outliers.

c Includes utilities and support services allocated by patient-days (electricity, water, gas, internet, taxes) aStudy sites as in Table 1. Data are not representative at the national level.

Among non-ICU admissions, mean direct medical costs ranged from USD PPP 3019–3189 in Argentina, USD PPP 624–676 in Brazil and USD PPP 1777–1974 in Colombia, with no statistically significant differences by RSV status within sites (bootstrap 95% CI of the mean difference including zero).

Intensive care unit admissions were consistently more costly than ward admissions across all sites, with mean direct medical costs approximately 2.5- to 6.7-fold higher admissions consistently more costly. Among RSV-positive cases, mean direct medical costs reached USD PPP 21,457 in Argentina, USD PPP 3080 in Brazil, and USD PPP 4912 in Colombia. In contrast, among RSV-negative ICU admissions, mean costs were USD PPP 11,771 in Argentina, USD PPP 2935 in Brazil, and USD PPP 6665 in Colombia, with no statistically significant differences between RSV-positive and RSV-negative groups at any site (bootstrap 95% CI of the mean difference including zero).

Across all settings, bed-day costs represented the largest share of direct medical costs, ranging from approximately 78% (ward) to 53% (ICU) in Argentina, 80% (ward) to 71% (ICU) in Brazil, and 84% (ward) to 74% (ICU) in Colombia, followed by respiratory support and medications.

### Direct non-medical costs per hospitalisation episode

Direct non-medical costs represented a smaller but meaningful component of total costs.

Among non-ICU admissions, mean values ranged from USD PPP 159–205 in Argentina, 212–225 in Brazil, and 376–419 in Colombia. These increased among ICU admissions, reaching USD PPP 420–558 in Argentina, 602–604 in Brazil, and 663–873 in Colombia. No statistically significant differences were observed according to RSV status in either setting, with the bootstrap 95% CI of the mean difference including zero in all comparisons. Operational overhead accounted for the largest proportion of direct non-medical costs, ranging from approximately 45% (ward) to 89% (ICU) in Argentina, 72% (ward) to 87% (ICU) in Brazil, and 53% (ward) to 67% (ICU) in Colombia.

### Indirect costs per hospitalisation episode

Indirect costs, reflecting caregiver productivity losses, were consistently observed across all sites but remained modest relative to direct medical costs. Among non-ICU admissions, mean indirect costs ranged from approximately USD PPP 50 to 200 per episode, representing a small proportion of total direct costs—around 2–3% in Argentina, 5–7% in Brazil, and up to 8–10% in Colombia.

In ICU admissions, indirect costs increased in absolute terms, in Brazil and Colombia, reaching mean values of USD PPP 395–426 per episode in Colombia, whereas in Argentina they remained similar to those observed in the ward. Even in this setting, indirect costs accounted for a limited share of total direct costs—below 1% in Argentina, approximately 2–3% in Brazil, and 5–8% in Colombia.

### Total societal costs and key cost drivers

**Fig. 1:**
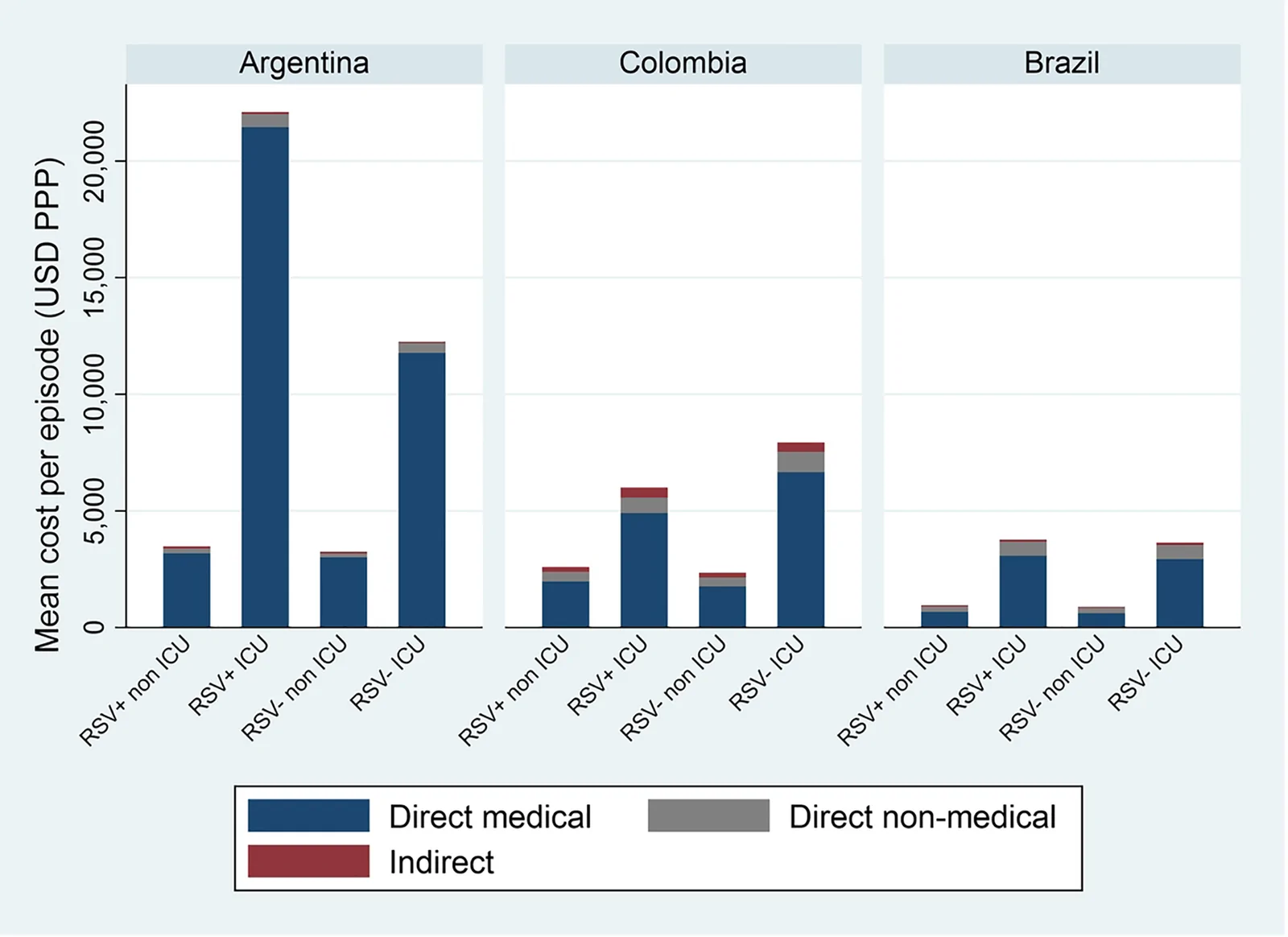
**Composition of average cost per hospitalisation episode by study site∗ and RSV status.** Bars represent the proportional contribution of direct medical, direct non-medical, and indirect costs to the total mean cost per episode. Costs are expressed in June 2025 purchasing power parity–adjusted US dollars (USD PPP). ICU, intensive care unit; RSV, respiratory syncytial virus. ∗Study sites: Hospital Militar Central Cosme Argerich (Buenos Aires, Argentina); Hospital Universitário da USP (São Paulo, Brazil); Hospital Militar Central (Bogotá, Colombia).

Total societal costs per hospitalisation episode are summarised in Table 3 and the composition of costs by component is shown in Fig. 1. Costs were consistently driven by ICU admission across all sites. ICU stays represented the main determinant of overall costs, with a several-fold increase compared with ward admissions regardless of RSV status. Across all study sites and levels of care, no comparison of mean costs between RSV-positive and RSV-negative episodes reached statistical significance (bootstrap 95% CI of the mean difference including zero), reinforcing that cost variation was driven by level of care rather than viral status. Intensive care unit (ICU) admissions were substantially more costly across all settings.

Mean total societal costs for ICU admissions reached USD PPP 22,099 (RSV-positive) and 12,253 (RSV-negative) in Argentina; 3770 and 3639 in Brazil; and 6002 and 7933 in Colombia, respectively. In contrast, non-ICU admissions were associated with substantially lower costs, (USD PPP 3250–3479 in Argentina, 885–959 in Brazil, and 2347–2593 in Colombia), and showed minimal differences between RSV-positive and RSV-negative patients within each site. The absolute difference in mean total societal cost per episode between RSV-positive and RSV-negative children was 1106 USD PPP (95% CI −477 to 3136) in Argentina, 489 USD PPP (95% CI −78 to 999) in Brazil, and 10 USD PPP (95% CI −633 to 628) in Colombia; in all three sites the confidence interval included zero, indicating no statistically significant difference by RSV status. Detailed cost components by study site and RSV status are presented in Supplementary Table S3. Results of the exchange-rate sensitivity analysis are presented in Supplementary Table S4.

### Length of stay and cost distribution

**Fig. 2:**
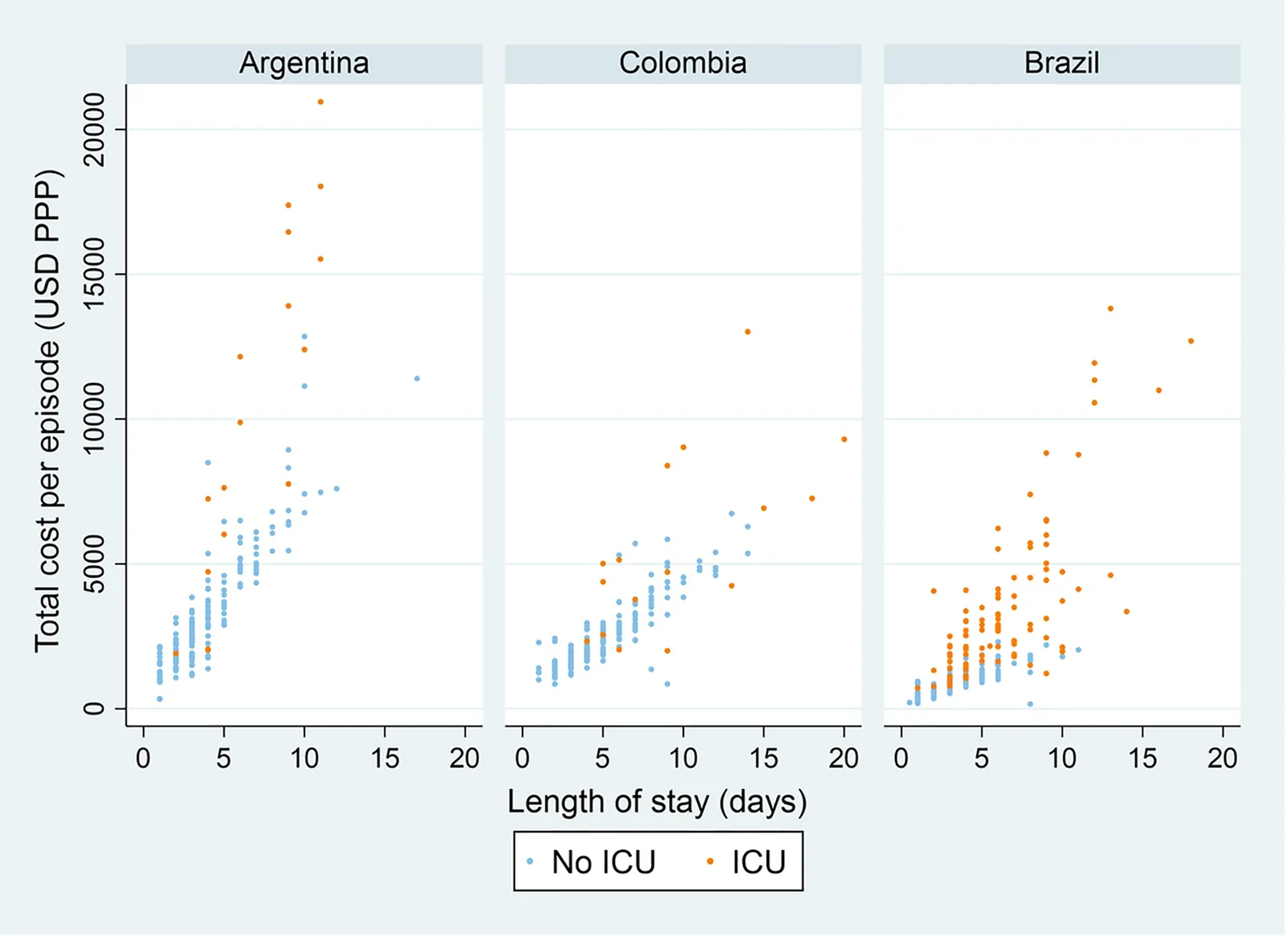
**Total cost per hospitalisation episode according to length of stay, by study site∗ and ICU admission status.** Scatter plots display total cost per hospitalisation episode (USD PPP) by length of stay (days), stratified by ICU admission status and country. Each point represents an individual hospitalisation episode. Total costs include direct medical, direct non-medical, and indirect costs. Extreme values above the 99th percentile for length of stay and total cost were excluded for visualisation purposes. ICU, intensive care unit; USD PPP, purchasing power parity–adjusted US dollars. ∗Study sites as in Fig. 1.

Fig. 2 shows the relationship between total cost and length of stay by study site and ICU status. In all settings, costs increased with longer stays, with a steeper gradient among ICU patients. ICU admission consistently shifted the cost distribution upward. Extreme values above the 99th percentile were excluded for visualisation.

### DALYs by study site and level of care (ward vs ICU)

**Fig. 3:**
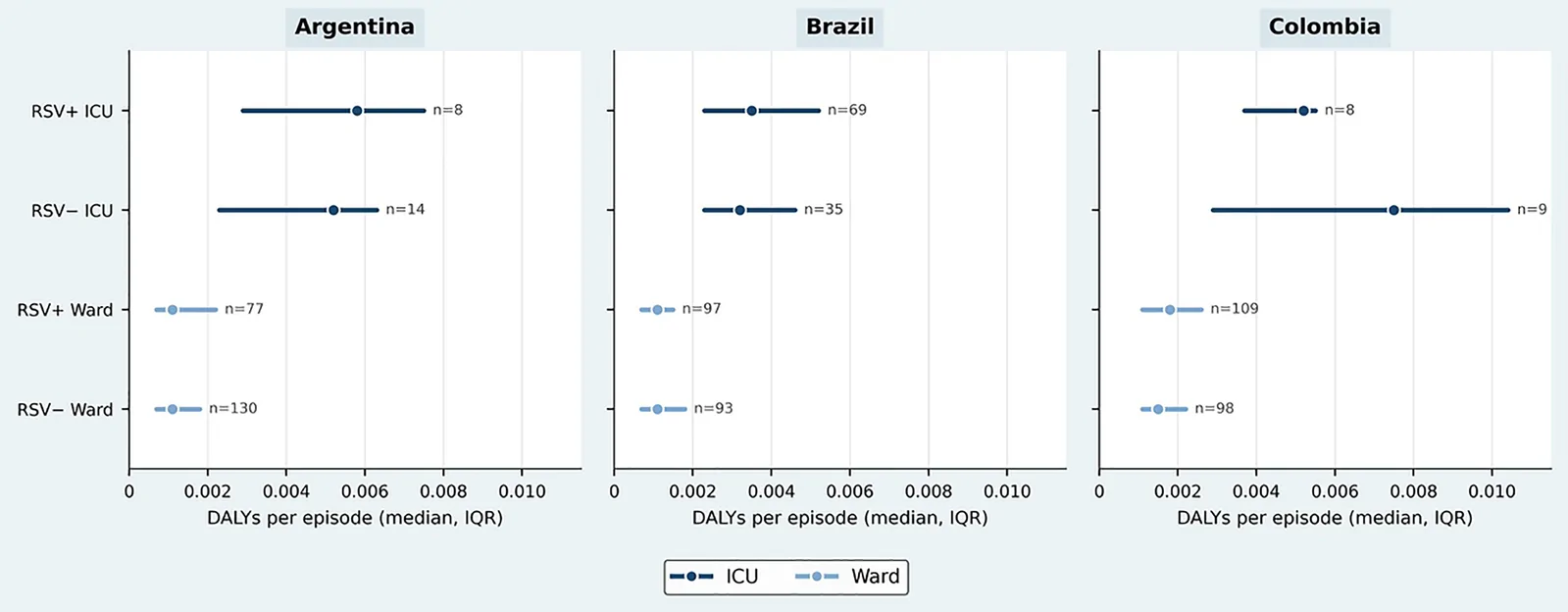
**DALYs per hospitalisation episode by study site∗, RSV status, and level of care.** Points represent median DALYs per episode and horizontal bars the interquartile range (P25–P75), stratified by RSV status and level of care (ward or intensive care unit) within each country. DALYs showed a highly right-skewed distribution driven by rare fatal events, and some strata included small numbers of patients; medians with interquartile ranges are therefore presented rather than means with confidence intervals. Sample sizes per subgroup are indicated adjacent to each estimate. DALY, disability-adjusted life year; ICU, intensive care unit; RSV, respiratory syncytial virus. ∗Study sites as in Fig. 1.

DALYs per episode were consistently higher among ICU compared with ward admissions across all sites (Fig. 3 and Supplementary Table S5).

In Argentina, median DALYs increased more than fivefold from 0.0011 (IQR 0.0007–0.0022) in RSV-positive ward patients to 0.0058 (0.0029–0.0075) in ICU cases, with similar patterns observed among RSV-negative patients. In Colombia, a comparable gradient was observed, with higher DALYs in ICU than ward admissions for both RSV-positive [0.0052 (0.0037–0.0055) vs 0.0018 (0.0011–0.0026)] and RSV-negative cases [0.0075 (0.0029–0.0104) vs 0.0015 (0.0011–0.0022)]. Notably, Colombia showed the highest DALY values overall, particularly among RSV-negative ICU patients. In Brazil, ward admissions were associated with low median DALYs (approximately 0.0011), while ICU cases showed a moderate increase [RSV+: 0.0035 (0.0023–0.0052); RSV–: 0.0032 (0.0023–0.0046)], though remaining lower than those observed in Argentina and Colombia. A single in-hospital death introduced marked right-skewness in this subgroup, substantially affecting mean estimates.

Overall, these findings indicate that level of care, rather than RSV status alone, is the primary determinant of DALYs per episode, with ICU admission consistently associated with a several-fold increase in health loss.

## Discussion

This study provides, to our knowledge, the first prospective, multicentre and standardised assessment of the clinical, economic and health burden of severe respiratory syncytial virus (RSV) disease among hospitalised children in three tertiary hospitals in Latin America. By integrating patient-level micro-costing with household out-of-pocket expenditures, productivity losses and disability-adjusted life years (DALYs), our analysis offers a comprehensive picture of the societal impact of RSV across three sites with distinct health system structures.

Although the burden of RSV in the region has been increasingly described, most available evidence in Latin America and other low- and middle-income settings is derived from cross-sectional or retrospective studies, frequently limited to direct medical costs. Recent regional syntheses confirm that economic data are sparse, methodologically heterogeneous, and rarely include non-medical or indirect costs, restricting cross-country comparability and policy relevance.^18^^,^^20^^,^^21^

Beyond costs, we incorporated DALYs as a complementary measure of health impact, which remains uncommon in hospital-based RSV studies in the region. Although mortality was rare, DALYs per episode were consistently higher among children requiring intensive care, reflecting the combined effect of longer hospital stays and more severe clinical courses. The joint evaluation of economic and health burden provides a unified framework for comparing RSV with other high-impact paediatric conditions.

Taken together, our findings suggest that severe RSV disease imposes a substantial clinical and economic burden even in middle-income health systems. Two patterns are particularly relevant for interpretation. First, total costs were driven primarily by the level and intensity of care rather than by viral aetiology: ICU admission was the dominant cost driver irrespective of RSV status, indicating that the economic weight of severe respiratory infection reflects the resources required to manage clinical severity rather than the specific pathogen. Second, this cost gradient was anchored in the hospital bed-day, which accounted for the largest share of direct medical costs across both ward and ICU settings, exceeding diagnostics, medications and respiratory support. By contrast, indirect costs from caregiver productivity losses, although consistently observed and increasing with severity, remained a minor component of total costs, so that direct medical expenditure—particularly ICU care—constituted the principal determinant of societal costs. The consistency of these patterns across three hospitals with distinct prices and financing arrangements suggests that they reflect structural features of severe RSV management rather than country-specific accounting. Importantly, the magnitude of costs varied widely across sites, reflecting differences in health system organisation, financing mechanisms and unit prices. Argentina consistently showed the highest absolute costs when expressed in USD PPP, followed by Colombia and Brazil. However, this cross-country gradient should be interpreted with caution. While PPP adjustment improves international comparability by accounting for differences in price levels, it may mask short-term macroeconomic distortions, particularly in high-inflation settings such as Argentina.^45^^,^^46^ In this context, PPP-based estimates may underestimate the current real cost of hospital care, whereas Brazil and Colombia, with more stable price environments, are likely to be better represented.

Our findings are consistent with the wide heterogeneity in RSV-associated hospitalisation costs reported across regions. In Colombia, Buendía et al. reported mean total costs per RSV episode of USD PPP 1060–1140, with ICU care costing three to four times more than ward care.^19^ Similar costs were described in additional Colombian studies using administrative and hospital-based data.^47^^,^^48^ Similar patterns have been described in Mexico^49^ and in Brazil, where ICU-managed cases exceeded USD PPP 10,000.^50^ These values are comparable to the upper range of previously published Latin American estimates summarised in the systematic review by Moreno et al., which reported costs per RSV hospitalisation episode between USD PPP 563 and 19,076 across Latin America.^21^ International evidence reinforces these findings and underscores the importance of a societal perspective. In Maputo, Mozambique, Rave et al. demonstrated that inpatient RSV episodes imposed a substantial burden on both health systems and households, with ICU costs approximately twice those of ward care.^15^ Similar patterns have been described in other low- and middle-income settings, where intensive care and prolonged hospitalisation remain the dominant cost drivers despite large differences in unit prices.^51^^,^^52^ Comparable severity gradients have also been observed in upper-middle- and high-income settings, where intensive care and prolonged hospitalisations remain the dominant cost drivers despite large differences in unit prices and health system organisation.^53^, ^54^, ^55^

This study has several important strengths. Its prospective, harmonised, multi-country design reduces key biases inherent to retrospective costing studies and enables direct comparison of utilisation patterns, clinical severity, and household costs across heterogeneous health systems. The use of patient-level micro-costing, combined with caregiver-reported non-medical and indirect costs, allows estimation from a true societal perspective rather than a payer-only viewpoint. In addition, the integration of DALYs alongside economic outcomes provides a complementary measure of health burden that remains uncommon in hospital-based RSV costing studies in Latin America and offers a unified framework for future cost-effectiveness and budget impact analyses of emerging preventive interventions.

Beyond quantifying current burden, our findings have direct implications for RSV prevention. Because per-episode costs and DALYs were driven mainly by hospitalisation and intensive care rather than by viral status, a substantial share of this burden corresponds to severe RSV-associated LRTI now potentially avoidable through recently licenced strategies—maternal RSVpreF vaccination and long-acting monoclonal antibodies such as nirsevimab and clesrovimab.^7^, ^8^, ^9^, ^10^, ^11^ As RSV accounted for approximately half of severe LRTI admissions, our patient-level estimates provide direct inputs for cost-effectiveness and budget impact analyses of these interventions across Latin American health systems.

Nevertheless, several limitations warrant careful consideration. The inclusion of a single tertiary referral hospital per country limits the generalisability of our findings, as health systems in Argentina, Brazil, and Colombia are highly complex and heterogeneous. Rather than pooling data across these structurally different systems—which may yield estimates difficult to interpret for any single country—we deliberately reported fully stratified, country-specific results, an approach recommended when between-country heterogeneity is substantial and decisions are made at the national level.^41^ Costs, care pathways, and severity profiles may differ substantially across regions, facility levels, and financing schemes, and differences in referral patterns and case-mix may have influenced resource use. This was most evident at the Brazilian site, where the substantially younger age of enrolled children (median 5.0 vs 17.0–18.0 months) and the centre's profile as a referral hospital admitting pre-triaged, more severely ill patients likely explain its higher rate of intensive care use. Additionally, in Brazil, within the public health system (Sistema Único de Saúde, SUS), costs may be underestimated, as healthcare financing is decentralised and shared across federal, state, and municipal levels; consequently, some expenditures may not be fully captured in hospital-level data and may instead be absorbed within institutional operational budgets. Unit costs and overhead allocations were partly derived from hospital administrative data and may reflect local accounting practices that are not fully comparable across institutions. Household expenditures and productivity losses were self-reported and therefore subject to recall bias; valuing time losses using minimum wages is a transparent and conservative approach but may not capture income heterogeneity or informal labour markets. In Argentina, patient recruitment spanned periods both before and after the introduction of maternal RSV vaccination; however, differences between these cohorts were not formally analysed. While this may have influenced clinical outcomes, any impact on cost estimates is likely to be limited, as no substantial cost differences were observed according to RSV status. Finally, although expressing costs in USD PPP improves cross-country comparability, PPP conversions may lag rapid macroeconomic changes—particularly in high-inflation settings such as Argentina—potentially masking short-term price distortions. To address this, we conducted a sensitivity analysis re-expressing costs at the official exchange rate (Supplementary Table S4). Because each country was represented by a single tertiary hospital located in a major urban centre, these estimates may not be fully generalisable to other regions, facility levels, or care-seeking patterns within each country. Race and ethnicity were not recorded and could not be analysed, further limiting assessment of representativeness. Together with the need for broader multi-site studies, these considerations should be considered when extrapolating results to inform policy decisions.

Taken together, severe RSV-associated lower respiratory tract infection imposed a substantial clinical, economic, and health burden among hospitalised children at the three participating centres in Argentina, Brazil, and Colombia. Across all sites, ICU admission emerged as the main determinant of both costs and DALYs, underscoring that disease severity and intensity of care—rather than RSV status alone—drive resource use and health loss. By integrating prospective patient-level micro-costing with household expenditures, productivity losses, and DALYs, this study provides a standardised societal assessment of RSV burden in three hospitals operating within distinct health system contexts. These findings offer, regionally relevant evidence to inform economic evaluations and support policy decisions regarding the prioritisation and implementation of emerging RSV preventive strategies in Latin America.

## Contributors

Conceptualisation, MTC, GG, RL, CP, PMI; methodology, MTC, GG; formal analysis, GG; investigation, JD, RA, TSc, TAS, LVS, AC, MPR, CW, RLC, GPM, CE, DSL, PS, PP; data curation, SC, GG; writing—original draft, GG; writing—review & editing, MTC, GG, RL, CP, PMI; supervision, MTC, RL, PMI; funding acquisition, MTC, RL, PMI.

GG, SC, and MTC directly accessed and verified the underlying data reported in this study. All authors had access to all the data in the study. MTC had full access to all the data and had final responsibility for the decision to submit for publication. All authors accept responsibility for the submission of this publication. All authors read and approved the final version of the manuscript.

## Data sharing statement

The datasets generated and/or analysed during the current study are not publicly available due to ethical and legal restrictions related to patient confidentiality. However, deidentified individual participant data underlying the results reported in this Article, together with a data dictionary, will be made available upon reasonable request. The study protocol and statistical analysis plan will also be available. Access will be granted to researchers who provide a methodologically sound proposal, subject to approval by the study investigators and the relevant institutional review boards, and after signing a data access agreement. Data sharing is subject to applicable institutional policies and regulations.

## Declaration of interests

GG, JD, PP, MP, SC, RA, TSc, MPR, CW, RLC, DSL, PS, and CE declare no competing interests. TAS reports institutional support from Fundación INFANT for the present study (funding provided to the institution, with no direct financial compensation to the author), and honoraria from Vertex Pharmaceuticals and travel support from Vertex Pharmaceuticals and the Bill & Melinda Gates Foundation, outside the submitted work. LVS reports grants from Vertex Pharmaceuticals, TimPel, and Fundación INFANT; consulting fees from Vertex Pharmaceuticals; honoraria for lectures from AstraZeneca, Vertex Pharmaceuticals, and Pfizer; and participation on an advisory board for Vertex Pharmaceuticals, all outside the submitted work. AC reports grants from AstraZeneca, Sanofi, Abbott, AbbVie, and ASCON; honoraria for lectures from AstraZeneca, Abbott, Sanofi, and AbbVie; and travel support from AstraZeneca, all outside the submitted work. CP reports that his organisation receives grant funding from the Bill & Melinda Gates Foundation related to RSV vaccines, outside the submitted work. GPM reports support from MSD for the present manuscript; grants or contracts from Pfizer, Boehringer Ingelheim, MSD, GSK, and Sanofi; consulting fees from Enanta and Pfizer; and honoraria for lectures from Pfizer and CSL Seqirus, outside the submitted work. PMI reports institutional funding from Merck & Company Inc. for the present study. RL reports institutional support from the Merck Investigator Studies Program for the present study, and participation on advisory boards for Pfizer and Merck, outside the submitted work. PMI reports institutional support from the Merck Investigator Studies Program (MISP-58738) (paid to Georgetown University) for the present study. MTC reports institutional support from the Merck Investigator Studies Program (MISP-58738) for the present study; institutional research grants from the Bill & Melinda Gates Foundation (INV-085205), the Merck Investigator Studies Program (MISP-102691), and Sanofi/AstraZeneca (RSV00105); and personal fees for consulting from MSD, honoraria for lectures from MSD, Sanofi, and Pfizer, and travel support from Sanofi and Pfizer, outside the submitted work.
