# Supplementary material for: Economic and health burden of severe respiratory syncytial virus infection among hospitalised children at three tertiary hospitals in Argentina, Brazil, and Colombia: a two-year prospective multicentre cohort study
Source: Lancet Reg Health Am. 2026 Aug 21;63:101601. doi: 10.1016/j.lana.2026.101601 (PMC13524657; doi:10.1016/j.lana.2026.101601)
Supplement: Supplementary file 1 [file mmc1.pdf]

Economic and health burden of severe respiratory syncytial virus infection among hospitalized children at three tertiary hospitals in Argentina, Brazil, and Colombia: a two-year prospective multicentre cohort study

Supplementary Material

Section	Page
1.Supplementary Methods	3
2. Supplementary Table 1. Number and proportion of missing observations for each analysed variable, by study site	8
3. Supplementary Table 2. Detailed resource utilization during hospitalization by study site and RSV status	10
4. Supplementary Table 3. Detailed societal cost (in 2025 PPP USD) per LRTI episode by study site and RSV status	14
5. Supplementary Table 4. Societal cost (in 2025 OER USD) per LRTI episode by study site and RSV status	22
6. Supplementary Table 5. DALYs per episode by study site and level of care	23
7. Supplementary Figure 1. Study participant flow diagram	24
8. Supplementary Figure 2. Monthly circulation of RSV-positive pediatric cases	25
9. References	26
10. Figure legends	26

1. **Supplementary Methods**

Biological Sample Collection and Viral Diagnostics

Nasopharyngeal swabs were obtained within 48 hours of admission using 3 mL of universal transport medium (UTM®, COPAN Diagnostics, Murrieta, CA, USA) and stored at –80°C until their processing. Samples were transported on dry ice to the Centro Infant de Medicina Traslacional laboratory in Buenos Aires at the end of the study's enrollment period. Viral detection was performed using real-time reverse transcriptase PCR (RT-PCR). For this purpose, RNA purification was first carried out using an automated magnetic particle transfer and processing system in a microplate format (MagMax-96 Express, Applied Biosystems), using 200 µL of a frozen aliquot. Subsequently, RT-PCR (StepOnePlus, Applied Biosystems) was performed to detect RSV (subgroups A and B) and other respiratory viruses (influenza, parainfluenza, metapneumovirus, adenovirus, rhinovirus, and SARS-CoV-2) using Applied Biosystems™ AgPath-ID™ One-Step RT-PCR Reagents. Human β-actin served as an internal control; samples without detectable β-actin on two independent assays were deemed inadequate and excluded. [1,2]

Sample size calculation

The minimum sample size required to estimate mean hospitalization costs was calculated using the following formula:

$$n = \left(\frac{\left(Z_{\frac{\alpha}{2}} * \ CV\right)}{d}\right)^2$$

where Z is the standard normal deviate for a two-sided 95% confidence level (1.96), CV is the coefficient of variation of costs, and d is the desired relative precision expressed as a proportion of the mean.

A coefficient variation of 0.5 and a relative precision of ±10% (d = 0.10) were assumed based on prior cost-of-illness studies of pediatric respiratory infections. This resulted in a minimum required sample of 292 hospitalized children. To account for seasonal variability in RSV circulation, a 10% inflation factor was applied, yielding a target sample size of 321 patients.

Currency conversion and valuation of productivity losses

All costs were initially estimated in local currency and converted to June 2025 international dollars using purchasing power parity (PPP) conversion factors obtained from the World Bank. Local currency values were first adjusted to June 2025 using official national consumer price indices.

Caregiver productivity losses were valued using the human capital approach. Caregivers reported the total number of working hours missed due to the child's hospitalization. These hours were monetized using country-specific statutory minimum wages, derived from official monthly minimum wages assuming 176 working hours per month (22 working days × 8 hours/day).

This conservative valuation approach was chosen to ensure transparency and comparability across countries and to avoid overestimation of indirect costs in heterogeneous labor markets**.**

Allocation of Overhead and Capital Costs

Direct medical costs were estimated using a micro-costing framework from the healthcare provider perspective. To avoid double counting, personnel salaries, capital equipment, and infrastructure were not costed separately when ward and pediatric intensive care unit (PICU) per-diem unit costs and procedure tariffs were used, as these unit costs were treated as full economic costs embedding staff, consumables, capital depreciation, and routine support services. This approach is consistent with international guidelines for economic evaluation and hospital costing. [3]

Operational overhead was therefore limited to non-clinical hospital services not explicitly captured in unit tariffs, including electricity, water, gas, telecommunications, information technology, administrative services, cleaning, security, and general facility maintenance. These costs were allocated using a top-down step-down method based on hospital patient-days, following standard hospital costing methodology. [3-6]

For each study site and calendar month, total hospital overhead expenditures were divided by the estimated number of inpatient days to obtain an overhead cost per patient-day. Because routinely collected patient-day statistics were not uniformly available across all sites, total patient-days were approximated from the number of licensed beds, average bed-occupancy rates, and days per month, separately for pediatric wards and the PICU. This approach has been widely used in settings where detailed hospital cost accounting systems are unavailable.[4,5]

To reflect the higher infrastructure intensity and energy consumption of critical care, PICU patient-days were weighted relative to general ward days using a multiplicative factor. Base-case analyses applied a PICU weight of 2.0, consistent with published estimates that intensive care units consume approximately 1.5–3 times more overhead resources per bed-day than general wards. [5-7]

Individual patient overhead costs were then calculated as:

$$
\begin{aligned}
&Overhead\ cost_i \\
&\qquad = (General\ ward\ days_i \times General\ ward\ overhead\ rate) \\
&\qquad + (PICU\ days_i \times PICU\ overhead\ rate)
\end{aligned}
$$

Uncertainty in overhead allocation was evaluated through one-way sensitivity analyses varying ward occupancy (60–80%), PICU occupancy (30–50%), and the PICU weighting factor (1.5–3.0).

Capital equipment was not costed separately in the base case because bed-day and procedure tariffs were treated as full economic costs incorporating equipment use and depreciation. Adding a separate capital cost component would risk double counting. [3]

Construction of Direct Medical Costs

Direct medical costs were calculated at the patient level using a bottom-up micro-costing approach. For each hospitalization episode, individual resource utilization was extracted from medical and administrative records, including:

- Ward and PICU bed-days
- Laboratory and microbiology tests
- Imaging studies

- Medications and intravenous therapies
- Respiratory support (oxygen, high-flow nasal cannula, non-invasive and invasive ventilation)
- Nutrition (enteral and parenteral)
- Procedures and monitoring

Each resource was multiplied by a country-specific unit cost obtained from hospital tariffs or accounting systems. Total direct medical cost per patient was calculated as the sum of all costed items:

$$Total\ direct\ medical\ cost_i = \sum_{\{j=1\}}^{\{J\}(Quantity_{\{ij\}} \times Unit\ cost_j)}$$

This patient-level summation allows heterogeneous patterns of care to be fully captured and is preferred over gross-costing methods in cost-of-illness studies of heterogeneous conditions such as severe LRTI. [3,8]

All direct medical costs were initially calculated in local currency and converted to June 2025 United States dollars adjusted for purchasing power parity (USD PPP) to enable cross-country comparability and reflect real resource use. PPP conversion is recommended in multinational economic evaluations when the objective is to compare economic burden rather than financial flows. [9-11]

Construction of Direct Non-Medical Costs

Direct non-medical costs were measured at the episode level from the caregiver perspective and included:

- Transportation to and from the hospital
- Meals purchased during hospitalization
- Accommodation (lodging near the hospital)
- Paid or hired caregivers
- Allocated hospital operational overhead

Caregiver-reported expenditures were obtained through structured interviews conducted during hospitalization. All reported costs were converted to USD PPP using country-specific conversion factors.
Total direct non-medical cost per episode was calculated as:

$$Direct\ non-medical\ cost_i = Transport_i + Meals_i + Lodging_i + Caregiver_i + Overhead_i$$

This approach aligns with the societal perspective recommended in burden-of-disease and cost-of-illness studies. [3,12]

Prevention of Double Counting

Several safeguards were implemented to avoid double counting across cost components. First, ward and PICU per-diem tariffs and procedure fees were assumed to embed personnel, capital, and routine operating costs. Therefore, no separate line items were added for staff salaries, medical equipment, or building depreciation. [3,8]

Second, overhead costs were limited to non-clinical services not already included in unit tariffs. Overhead was allocated using patient-days rather than added as a flat hospital-level expenditure, ensuring proportional attribution without duplication.

Third, when constructing total direct costs, direct medical and direct non-medical components were summed only after all individual items were costed once and only once.

Statistical Handling of Costs and Uncertainty

Cost data were highly right-skewed, as is typical for hospitalization costs. Therefore, all mean cost estimates were accompanied by non-parametric bootstrap confidence intervals. For each country and clinical severity group (Country × Group4), 1,000 bootstrap resamples were drawn with replacement from the original patient-level data. The mean cost was recalculated for each resample, and the 2.5th and 97.5th percentiles of the empirical distribution were used to construct 95% confidence intervals. [13,14]

Bootstrap resampling was applied to direct medical, direct non-medical, and total direct costs, and was restricted to strata with at least five patients incurring non-zero costs, consistent with recommended practice for stable variance estimation.

All analyses were conducted using Stata version 15 (StataCorp, College Station, TX).

Caregiver Interview Questionnaire (Household Costs Related to Hospitalization)

The following structured questionnaire was administered to caregivers during hospitalization to capture household out-of-pocket expenditures and productivity losses related to the index episode (English translation; original administered in Spanish/Portuguese).

Questions

- How long did it take to reach the first place of medical care from your home? (minutes)
- Approximately how far is your home from the place of care? (kilometers)
- How did you and the patient travel to the place of care? (Walking/bicycle; Subway/metro/train [+ ticket cost]; Bus [+ ticket cost]; Taxi/rideshare [+ trip cost]; Car/motorcycle [+ parking cost]; Other [specify + cost])
- Total transportation expenditure related to the hospitalization during the days of admission (including fuel, parking, tickets, and tolls, as applicable)
- Was the patient referred to the hospital by ambulance?
- If yes, did you have to pay for the ambulance? How much?
- Who accompanied the child to the place of care? (Mother; Father; Other relative; Other)
- Did the accompanying person(s) have to miss work to accompany the child?
- If yes, how many total hours were missed during the days of hospitalization?
- Was any pay deducted due to the absence?
- If yes, what was the total amount deducted?
- Did any accompanying person incur non-healthcare-related expenses during the hospitalization (food, drink, diapers, other)?
- If yes, what was the approximate total expense per day of hospitalization? (currency: ARS/COP/BRL)

- Did the accompanying person stay with the child at the place of care during hospitalization?
- If not, did they stay elsewhere (e.g., hotel, guesthouse, other lodging)?
- If yes, what was the cost per day, and for how many days?
- If there were other children at home, were they left in the care of a paid caregiver?
- If yes, what was the cost per day and the number of days?
- Did you have to pay for any hospital fee/co-payment, procedure, or medication during the hospitalization? (Hospital fee/co-payment; Medications [specify + cost]; Diagnostic studies [specify + cost]; Procedures [specify + cost])
- Did you have any consultation related to the current episode prior to hospitalization?
- If yes, how many times?
- What was the cost of one consultation?
- How did you and the patient travel to the place of care for this consultation? (same transport categories and costs as Q26)
- Did you have to pay for any hospital fee/co-payment, procedure, or medication during these visits? (Medications; Hospital fee; Diagnostic studies; Procedures — each with item specified and cost)

2. **Supplementary Table 1. Number and proportion of missing observations for each analysed variable, by study site***

Variable	Argentina (n=229)	Brazil (n=294)	Colombia (n=224)	Total (n=747)
**Exposure**				
RSV status (RT-PCR)	1 (0.4%)	2 (0.7%)	0 (0.0%)	3 (0.4%)
**Demographic characteristics**				
Age	0 (0.0%)	0 (0.0%)	0 (0.0%)	0 (0.0%)
Sex	0 (0.0%)	0 (0.0%)	0 (0.0%)	0 (0.0%)
**Clinical characteristics**				
Prematurity	0 (0.0%)	0 (0.0%)	2 (0.9%)	2 (0.3%)
Comorbidities	0 (0.0%)	0 (0.0%)	1 (0.4%)	1 (0.1%)
Prior LRTI-related hospitalisation	1 (0.4%)	0 (0.0%)	0 (0.0%)	1 (0.1%)
Presenting symptoms	0 (0.0%)	0 (0.0%)	1 (0.4%)	1 (0.1%)
**Disease course and outcomes**				
Total length of hospital stay	0 (0.0%)	0 (0.0%)	0 (0.0%)	0 (0.0%)
ICU admission	0 (0.0%)	1 (0.3%)	0 (0.0%)	1 (0.1%)
ICU length of stay	1 (0.4%)	0 (0.0%)	0 (0.0%)	1 (0.1%)
Respiratory support modality (oxygen, HFNC, NIV, IMV)	2 (0.9%)	0 (0.0%)	1 (0.4%)	3 (0.4%)
Duration of mechanical ventilation	1 (0.4%)	0 (0.0%)	1 (0.4%)	2 (0.3%)
Outcome at discharge / in-hospital mortality	0 (0.0%)	0 (0.0%)	2 (0.9%)	2 (0.3%)
**Healthcare resource utilisation**				
Medication use (antibiotics, corticosteroids, bronchodilators, analgesics, vasoactive drugs, sedation-analgesia, diuretics)	0 (0.0%)	0 (0.0%)	0 (0.0%)	0 (0.0%)
Imaging studies	1 (0.4%)	1 (0.3%)	0 (0.0%)	2 (0.3%)
Laboratory tests	0 (0.0%)	0 (0.0%)	0 (0.0%)	0 (0.0%)
Microbiological cultures	0 (0.0%)	0 (0.0%)	0 (0.0%)	0 (0.0%)
**Household and indirect costs (caregiver interview)**				
Caregiver interview not completed	3 (1.3%)	2 (0.7%)	2 (0.9%)	7 (0.9%)
Transportation	2 (0.9%)	1 (0.3%)	0 (0.0%)	3 (0.4%)

Variable	Argentina (n=229)	Brazil (n=294)	Colombia (n=224)	Total (n=747)
Meals	0 (0.0%)	1 (0.3%)	0 (0.0%)	1 (0.1%)
Accommodation	0 (0.0%)	0 (0.0%)	0 (0.0%)	0 (0.0%)
Paid caregiver	0 (0.0%)	0 (0.0%)	0 (0.0%)	0 (0.0%)
Caregiver employment status	1 (0.4%)	0 (0.0%)	0 (0.0%)	1 (0.1%)
Days of work lost	1 (0.4%)	0 (0.0%)	1 (0.4%)	2 (0.3%)

Data are n (%) of participants with missing values for each variable.. Complete-case analysis was applied and no imputation was performed. Zero values in household cost items reflect that no expenditure was incurred and are not counted as missing.

*Study sites as in Table 1. Data are not representative at the national level.

HFNC, high-flow nasal cannula; ICU, intensive care unit; IMV, invasive mechanical ventilation; LRTI, lower respiratory tract infection; NIV, non-invasive ventilation; RSV, respiratory syncytial virus; RT-PCR, reverse transcription polymerase chain reaction.

3. Supplementary Table 2. Detailed Resource utilization during hospitalization among children younger than five years with LRTI, by study sites.

	ARG RSV (+) non ICU	ARG RSV (–) non ICU	p value	ARG RSV (+) ICU	ARG RSV (-) ICU	p value	BRL RSV (+) non ICU	BRL RSV (–) non ICU	p value	BRL RSV (+) ICU	BRL RSV (-) ICU	p value	COL RSV (+) non ICU	COL RSV (–) non ICU	p value	COL RSV (+) ICU	COL RSV (-) ICU	p value
**Total n (%)**	77 (37.1)	130 (62.9)		8 (36.4)	14 (63.6)		97 (51.1)	93 (48.9)	.	69 (72.7)	35 (27.3)		109 (52.7)	98 (47.3)	.	8 (47.0)	9 (53.0)	
**Any antibiotic n (%)**	25/77 (32.5)	30/130 (23.1)	0.15	7/8 (87.5)	10/14 (71.4)	0.37	20/97 (20.6)	24/93 (25.8)	.40	42/69 (60.9)	22/35 (62.9)	.84	17/109 (15.6)	24/98 (24.5)	.09	4/8 (50.0)	5/9 (55.6)	0.80
**Ceftriaxone n (%)**	5/77 (6.5)	6/130 (4.6)	0.38	4/8 (50)	2/14 (14.3)	0.10	5/97 (5.2)	4/93 (4.3)	.53	22/69 (31.9)	13/35 (37.1)	.37	0	0	>0.99	0	2/9 (22.2)	0.27
**Meropenem n (%)**	0	0	-	1/8 (12.5)	1/14 (7.1)	0.60	0	0	-	1/69 (1.2)	1/35 (2.9)	.56	0	0	-	0	0	-
**Ampicilin n (%)**	5/77 (6.5)	7/130 (5.4)	0.77	0	2/14 (14.3)	0.18	0	1/93 (1.1)	.31	1/69 (1.5)	1/35 (2.9)	.62	0	0	>0.99	0	1/9 (11.1)	0.29
**Amoxycilin n (%)**	15/77 (19.5)	22/130 (16.9)	0.68	1/8 (12.5)	0	0.40	10/97 (10.3)	12/93 (12.9)	.65	12/69 (17.4)	7/35 (20.0)	.63	14/109 (12.8)	17/98 (17.4)	.49	0	0	-
**Amxycilin-clavulanic n (%)**	0	2/130 (1.5)	0.50	0	1/14 (7.1)	0.55	6/97 (6.2)	11/93 (11.8)	.23	18/69 (26.1)	10/35 (28.6)	.61	0	0	-	0	1/8 (12.5)	0.29
**Claritromicin n (%)**	3/77 (3.9)	7/130 (5.4)	0.75	4/8 (50.0)	5/14 (35.7)	0.69	0	0	-	1/69 (1.45)	0	.39	0	2/98 (2.0)	.22	3/8 (37.5)	0	-
**Ampicilin-sulbactam (%)**	1/77 (1.3)	1/130 (0.8)	>0.99	1/8 (12.5)	4/14 (28.6)	0.32	2/97 (2.1)	1/93 (1.1)	>0.99	3/69 (4.4)	3/35 (8.6)	.67	0	0	-	0	0	-
**Cefotaxime (%)**	0	0	-	0	0	-	1/97 (1.0)	0	>0.99	0	1/35 (2.9)	.38	0	0	-	0	0	-
**Clindamicin n (%)**	1/77 (1.3)	2/130 (1.5)	>0.99	1/8 (12.50)	4/14 (28.57)	0.61	0	0	-	0	0	-	0	0	-	0	0	-
**Piperaacilin-tazobactam n (%)**	0	0	-	2/8 (25.0)	1/14 (7.1)	0.54	0	0	-	0	0	-	0	0	-	0	0	-
**Gentamicin n (%)**	0	0	-	0	0	-	0	01/93 (1.1)	>0.99	0	0	-	0	1/98 (1.0)	.47	0	1/9 (11.1)	0.50
**Amikacin n (%)**	0	0	-	0	0	-	1/97 (1.0)	0	.31	2/69 (2.9)	0	.17	0	0	-	0	0	-
**Penicilin n (%)**	1/77 (1.3)	5/130 (3.9)	0.41	1/8 (12.5)	0	0	0	3/93 (3.2)	.25	0	0	-	0	2/98 (2.0)	.23	0	0	-
**Vancomicin n (%)**	0	0	-	2/8 (25)	0	0.11	0	0	-	4/69 (5.8)	2/35 (5.7)	.69	0	0	-	0	1/9 (11.1)	>0.99
**Oseltamivir n (%)**	17/77 (22.1)	18/130 (13.8)	0.13	3/8 (37.5)	6/14 (42.9)	>0.99	1/97 (1.0)	2/93 (2.2)	.62	0	0	-	1/109 (0.9)	6/98 (6.1)	.05	1/8 (12.5)	1/9 (11.1)	>0.99
**Any corticosteroid n (%)**	24/77 (31.1)	54/130 (41.5)	0.18	7/8 (87.5)	13/14 (92.9)	0.59	43/97 (44.3)	44/93 (47.3)	.64	26/69 (37.7)	14/35 (40.0)	.74	42/109 (38.5)	33/98 (33.7)	.38	6/8 (75.0)	6/9 (66.7)	>0.99
**metilrednisolone n (%)**	17/77 (22.1)	33/130 (25.5)	0.70	7/8 (87.5)	8/14 (57.1)	0.17	0	0	-	0	0	-	0	0	-	0	0	-
**Betametasone n(%)**	3/77 (3.9)	3/130 (2.3)	0.67	1/8 (12.5)	0	0.33	0	0	-	0	0	-	0	0	-	0	0	-
**Hydrocortisone n (%)**	6/77 (7.8)	7/130 (5.4)	0.58	0	8/14 (57.1)	**.005**	0	0	-	0	0	-	0	0	-	0	0	-
**Dexamethasone n**	12/77 (15.6)	26/130 (20.0)	0.35	6/8	10/14	0.63	0	1/93 (1.1)	.49	10/69	1/35 (2.9)	**.037**	6/109 (5.50)	1/98 (1.0)	.12	1/8 (12.5)	2/9	>0.99

	ARG RSV (+) non ICU	ARG RSV (–) non ICU	p value	ARG RSV (+) ICU	ARG RSV (-) ICU	p value	BRL RSV (+) non ICU	BRL RSV (–) non ICU	p value	BRL RSV (+) ICU	BRL RSV (-) ICU	p value	COL RSV (+) non ICU	COL RSV (–) non ICU	p value	COL RSV (+) ICU	COL RSV (-) ICU	p value
(%)				(75.0)	(71.4)					(14.5)							(22.2)	
**Any brochodilator n (%)**	70/77 (90.9)	113/130 (86.9)	0.53	7/8 (87.5)	14/14 (100)	>0.99	65/97 (67.0)	66/93 (71.0)	0.59	39/69 (56.5)	19/35 (54.3)	0.85	95/109 (87.2)	63/98 (64.3)	**<0.0001**	7/8 (87.5)	6/9 (66.7)	0.58
**Albuterol n (%)**	70/77 (90.9)	113/130 (86.9)	0.53	7/8 (87.5)	14/14 (100)	>0.99 00	65/97 (67.0)	65/93 (69.9)	0.59	38/69 (55.1	19/35 (54.3	0.85	87/109 (79.8)	61/98 (62.2)	.001	7/8 (87.5)	5/9 (55.6)	0.58
**Ipratropuim bromide n (%)**	20/77 (26.0)	48/130 (36.9)	0.12	5/8 (62.5)	8/14 (57.1)	>0.99	6/97 (6.2)	7/93 (7.5)	0.78	6/69 (8.7)	4/35 (11.4)	0.74	13/109 (11.9)	2/98 (2.0	.003	3/8 (37.5)	2/9 (22.2)	0.62
**Hypertonic saline n (%)**	0	0	-	0	0	-	3/97 (3.1)	5/93 (5.4)	0.51	1/69 (1.4)	2/35 (5.7)	0.29	17/109 (15.6)	9/98 (9.2)	.15	1/8 (12.5)	1/9 (11.1)	>0.99
**Budesonide n (%)**	0	4/130 (3.1)	0.15	0	1/14 (7.1)	>0.99	0	0	-	6/69 (8.7)	0	0.17	13/109 (11.9)	8/98 (8.2)	.36	3/8 (37.5)	2/9 (22.2)	0.62
**Fluticasone n (%)**	4/77 (5.2)	8/130 (6.2)	0.78	0	3/14 (21.4)	0.26	0	0	-	0	0	-	4/109 (3.7)	2/98 (2.0)	.69	0	0	-
**Any analgesic n (%)**	9/77 (11.7)	11/130 (8.5)	0.49	5/8 (62.5)	3/14 (21.4)	0.07	58/97 (59.8)	51/93 (54.8)	0.52	58/69 (84.1)	26/35 (74.3)	0.26	43/109 (39.4)	36/98 (36.7)	.70	6/8 (75.0)	4/9 (44.4)	0.19
**Acetaminophen n (%)**	1/77 (1.3)	1/130 (0.8)	>0.99	1/8 (12.5)	0	0.33	20/97 (20.6)	13/93 (14.0)	0.21	24/69 (34.8)	14/35 (40.0)	0.65	43/109 (39.4)	34/98 (34.7)	.50	5/8 (62.5)	4/9 (44.4)	0.48
**Ibuprofen n (%)**	6/77 (7.8)	3/130 (2.3)	0.06	0	0	-	0	0	-	3/69 (4.3)	0	0.56	4/109 (3.7)	3/98 (3.1)	>0.99	0	0	-
**Dypirone n (%)**	3/77 (3.9)	8/130 (6.2)	0.55	5/8 (62.5)	3/14 (21.4)	0.07	43/97 (44.3)	42/93 (45.2)	0.90	51/69 (73.9)	24/35 (68.6)	0.57	3/109 (2.8)	7/98 (7.1)	.13	3/8 (37.5)	1/9 (11.1)	0.24
**Any vasoactive drug n (%)**	0	0	-	3/8 (37.5)	2/14 (14.3)	0.30	3/97 (3.1)	3/93 (3.2)	>0.99	14/69 (20.3)	6/35 (17.1)	0.22	1/109 (0.9)	2/98 (2.0)	.57	1/8 (12.5)	1/9 (11.1)	>0.99
**Adrenaline n (%)**	0	0	-	0	0	-	3/97 (3.1)	3/93 (3.2)	>0.99	14/69 (20.3)	6/35 (17.1)	0.22	1/109 (0.9)	2/98 (2.0)	.57	1/8 (12.5)	1/9 (11.1)	>0.99
**Dopamine n (%)**	0	0	-	0	0	-	0	0	-	0	0	-	0	0	-	0	0	-
**Milrinone n (%)**	0	0	-	1/8 (12.5)	0	0.27	0	0	-	0	2/35 (5.7)	0.13	0	0	-	0	0	-
**Dobutamine n (%)**	0	0	-	0	0	-	0	0	-	1/69 (1.4)	0	0.33	0	0	-	0	0	-
**Noradrenaline n (%)**	0	0	-	2/8 (25.0)	2/14 (14.3)	-	3/97 (3.1)	3/93 (3.2)	>0.99	14/69 (20.3)	6/35 (17.1)	0.79	1/109 (0.9)	2/98 (2.0)	.58	1/8 (12.5)	1/9 (11.1)	>0.99
**Any sedation-analgesia n %)**	1/77 (1.3)	0	0.36	6/8 (75.0)	7/14 (50.0)	0.22	1/97 (1.0)	0	0.50	37/69 (53.6)	22/35 (62.9)	0.35	0	0	-	1/8 (12.5)	1/9 (11.1)	>0.99
**Midazolam n (%)**	0	0	-	4/8 (50.0)	4/14 (28.6)	0.27	1/97 (1.0)	0	0.50	14/69 (20.3)	12/35 (34.3)	0.13	0	0	-	0	0	-
**Fentanyl n (%)**	0	0	-	5/8 (62.5)	5/14 (35.7)	0.24	0	0	-	15/69 (21.7)	5/35 (14.3)	0.35	0	0	-	0	0	-
**Dexmedetomidine n (%)**	1/77 (1.3)	0	0.36	5/8 (62.5)	6/14 (42.9)	0.31	1/97 (1.0)	0	0.50	35/69 (50.7)	20/35 (57.1)	0.53	0	0	-	0	1/9 (11.1)	0.32
**Ketamine n (%)**				3/8 (37.5)	4/14 (28.6)	0.66	1/97 (1.0)	0	0.50	17/69 (24.6)	14/35 (40.0)	0.11	0	0	-	1/8 (12.5)	0	0.31
**Any diuretic n (%)**	0	0	-	4/8 (50.0)	6/14 (42.9)	0.74	1/97 (1.0)	0	0.30	11/69 (15.9)	4/35 (11.4)	0.54	1/109 (0.9)	0	.32	0	2/9 (22.2)	0.47
**Furosemide n (%)**	0	0	-	4/8 (50.0)	4/14 (28.6)	0.33	1/97 (1.0)	0	0.30	10/69 (14.5)	4/35 (11.4) 0.6	0.77	1/109 (0.9)	0	.32	0	2/9 (22.2)	0.47

	ARG RSV (+) non ICU	ARG RSV (–) non ICU	p value	ARG RSV (+) ICU	ARG RSV (-) ICU	p value	BRL RSV (+) non ICU	BRL RSV (–) non ICU	p value	BRL RSV (+) ICU	BRL RSV (-) ICU	p value	COL RSV (+) non ICU	COL RSV (–) non ICU	p value	COL RSV (+) ICU	COL RSV (-) ICU	p value
Spironolactone n (%)	0	0	-	0	2/14 (14.3)	0.50	0	0	-	5/69 (7.2)	0	0.16	0	0	-	0	0	-
Enteral nutrition n (%)	4/77 (5.2)	1/130 (0.8)	0.08	1/8 (12.5)	2/14 (14.3)	>0.99	12/97 (12.4)	6/93 (6.5)	0.15	55/69 (79.7)	24/35 (68.6)	0.19	0	0	-	0	0	-
Hydration IV n (%)	9/77 (11.7)	8/130 (6.2)	0.17	5/8 (62.5)	9/14 (64.3)	0.91	3/97 (3.1)	4/93 (4.3)	0.72	29/69 (42.0)	14/35 (40.0)	0.84	6/109 (5.5)	9/98 (9.2)	0.28	2/8 (25.0)	2/9 (22.2)	0.91
Parenteral Nutrition use n (%)	0	0	-	1/8 (12.5)	-	0.24	0	0	-	0	0	-	0	0	-	0	0	-
Any imaging n (%)	52/77 (67.5)	77/130 (59.2)	0.24	8/8 (100)	13/14 (92.8)	0.39	65/97 (67.0)	71/93 (76.3)	0.16	69/69 (100)	35/35	>0.99	92/109 (84.4)	84/98 (85.7)	0.81	8/8 (100)	9/9 (100)	>0.99
Radiography n (%)	52/77 (67.5)	77/130 (59.2)	0.24	8/8 (100)	13/14 (92.8)	0.39	65/97 (67.0)	71/93 (76.3)	0.16	69/69 (100)	35/35 (100)	>0.99	89/109 (81.6)	84/98 (85.7)	0.45	8/8 (100)	9/9 (100)	>0.99
Ultrasound n (%)	0	5/130 (3.8)	0.11	2/8 (25.0)	1/14 (7.1)	0.22	3/97 (3.1)	3/93 (3.2)	0.97	3/69 (4.3)	4/35 (11.4)	0.19	0	0	-	0	2/9 (22.2)	0.14
Computed Tomography (CT) n (%)	0	1/130 (0.7)	0.42	0	1/14 (7.1)	0.36	1/97 (1.0)	0	0.32	7/69 (10.1)	6/35 (17.1)	0.34	1/109 (0.9)	0	0.31	0	2/9 (22.2)	0.14
Magnetic Resonance Imaging (MRI) n (%)	0	0	-	0	0	-	0	0	-	0	0	-	0			0	1/9 (11.1)	0.29
Electrocardiogram (ECG) n (%)	0	0	-	1/8 (12.5)	0	>0.99	0	0	-	0	0	-	1/109 (0.9)	1/98 (1.0)	>0.99	0	0	-
Echocardiography n (%)	3/77 (3.9)	1/130 (0.8)	0.12	2/8 (25.0)	1/14 (7.1)	0.27	1/97 (1.0)	0	0.32	4/69 (5.8)	2/35 (5.7)	>0.99	12/109 (11.0)	11/98 (11.2)	>0.99	2/8 (25.0)	2/9 (22.2)	>0.99
Any laboratory test n (%)	6/77 (7.8)	20/130 (15.4)	0.11	7/8 (87.5)	9/14 (64.3)	0.24	96/97 (99.0)	91/93 (97.8)	0.54	69/69 (100.0)	35/35 (100.0)	>0.99	105/109 (96.3)	98/98 (100.0)	0.06	8/8 (100.0)	9/9 (100.0)	>0.99
Complete Blood Count (CBC) n (%)	6/77 (7.8)	19/130 (14.6)	0.18	7/8 (87.5)	9/14 (64.3)	0.35	21/97 (21.6)	15/93 (16.1)	0.38	52/69 (75.4)	28/35 (80.0)	0.79	32/109 (29.4)	32/98 (32.6)	0.64	7/8 (87.5)	8/9 (88.9)	>0.99
Coagulation profile n (%)	0	2/130 (1.5)	>0.99	4/8 (50.0)	5/14 (35.7)	0.68	1/97 (1.0)	1/93 (1.1)	>0.99	10/69 (14.5)	7/35 (20.0)	0.60	1/109 (0.9)	1/98 (1.0)	>0.99	0	1/9 (11.1)	>0.99
Hepatic profile n (%)	1/77 (1.3)	9/130 (6.9)	0.10	7/8 (87.5)	7/14 (50.0)	0.09	6/97 (6.2)	5/93 (5.4)	>0.99	11/69 (15.9)	8/35 (22.9)	0.45	2/109 (1.8)	2/98 (2.0)	>0.99	0	2/9 (22.2)	0.18
Renal function n (%)	3/77 (3.9)	15/130 (11.5)	0.06	6/8 (75.0)	7/14 (50.0)	0.25	11/97 (11.3)	3/93 (3.2)	0.04	47/69 (68.1)	28/35 (80.0)	0.23						
Erythrocyte Sedimentation Rate n (%)	0	2/130 (1.5)	0.55	0	0	-	0	0	-	1/69 (1.4)	0	>0.99	1/109 (0.9)	0	>0.99	0	0	-
C-reactive protein (PCR) n (%)	4/77 (5.2)	8/130 (6.2)	>0.99	7/8 (87.5)	7/14 (50.0)	0.18	19/97 (19.6)	13/93 (14.0)	0.35	50/69 (72.5)	28/35 (80.0)	0.39	23/109 (21.1)	24/98 (24.5)	0.67	5/8 (62.5)	9/9 (100.0)	0.11
Blood gases n (%)	1/77 (1.3)	2/130 (1.5)	>0.99	4/8 (50.0)	4/14 (28.6)	0.39	8/97 (8.2)	2/93 (2.2)	0.05	42/69 (60.9)	21/35 (60.0)	0.93	1/109 (0.9)	0	>0.99	0	4/9 (44.4)	0.09
Ionogram n (%)	4/77 (5.2)	9/130 (6.9)	0.76	6/8 (75.0)	6/14 (42.9)	0.21	8/97 (8.2)	3/93 (3.2)	0.12	44/69 (63.8)	26/35 (74.3)	0.28	11/109 (10.1)	8/98 (8.2)	0.66	4/8 (50.0)	5/9 (55.6)	0.82

	ARG RSV (+) non ICU	ARG RSV (–) non ICU	p value	ARG RSV (+) ICU	ARG RSV (-) ICU	p value	BRL RSV (+) non ICU	BRL RSV (–) non ICU	p value	BRL RSV (+) ICU	BRL RSV (-) ICU	p value	COL RSV (+) non ICU	COL RSV (–) non ICU	p value	COL RSV (+) ICU	COL RSV (-) ICU	p value
**Glucose n (%)**	1/77 (1.3)	8/130 (6.2)	0.09	5/8 (62.5)	5/14 (35.7)	0.25	0	0	-	1/69 (1.4)	0	0.47	2/109 (1.8)	4/98 (4.1)	0.30	1/8 (12.5)	2/9 (22.2)	0.60
**Urinalysis n (%)**	1/77 (1.3)	0	0.32	1/8 (12.5)	0	0.24	7/97 (7.2)	5/93 (5.4)	0.63	24/69 (34.8)	6/35 (17.1)	0.06	3/109 (2.8)	4/98 (4.1)	0.57	0	1/9 (11.1)	0.31
**Any culture n (%)**	11/77 (14.3)	9/130 (6.9)	0.08	6/8 (75.0)	8/14 (57.1)	0.40	12/97 (12.3)	11/93 (11.8)	0.90	45/69 (65.2)	21/35 (60.0)	0.60	3/109 (2.8)	4/98 (4.1)	0.57	3/8 (37.5)	3/9 (33.3)	0.86
**Hemoculture n (%)**	10/77 (13.0)	9/130 (6.9)	0.32	6/8 (75.0)	8/14 (57.1)	0.64	9/97 (9.3)	8/93 (8.6)	>0.99	41/69 (59.4)	20/35 (57.1)	0.65	3/109 (2.8)	4/98 (4.1)	0.72	2/8 (25.0)	3/9 (33.3)	>0.99
**Urine Culture n (%)**	0	1/130 (0.8)	>0.99	4/8 (50.0)	3/14 (21.4)	0.31	6/97 (6.2)	3/93 (3.2)	0.50	23/69 (33.8)	7/35 (20.0)	0.24	1/109 (0.9)	1/98 (1.0)	>0.99	0	1/9 (11.1)	0.29
**Cerebrospinal Fluid (CSF) Culture n (%)**	0	0	-	0	0	-	0	0	-	1/69 (1.4)	1/34 (2.9)	>0.99	0	0	-	0	0	-
**Pleural Fluid Culture n (%)**	0	0	-	0	1/14 (7.1)	0.27	1/97 (1.0)	0	>0.99	1/69 (1.4)	3/35 (8.6)	0.15	0	0	-	0	2/9 (22.2)	0.08
**Tracheal Aspirate Culture n (%)**	0	0	-	2/8 (25.0)	4/14 (28.6)	>0.99	0	0	-	15/69 (21.7)	5/35 (14.3)	0.42	0	0	-	1/8 (12.5)	0	0.47
**Stool Culture n (%)**	1/77 (1.3)	0	0.38	0	0	-	0	0	-	3/69 (4.3)	3/35 (8.6)	0.41	0	0	-	0	0	-
**Peritoneal dialysis n (%)**	0	0	-	0	0	-	0	0	-	0	1/35 (2.9)	>0.99	0	0	-	0	0	-
**Any oxygen requirement n (%)**	76/77 (98.7)	126/130 (96.9)	>0.99	8/8 (100)	14/14 (100)	-	91/97 (93.8)	83/93 (89.2)	0.40	69/69 (100.0)	34/35 (97.1)	0.34	107/109 (98.2)	96/98 (98.0)	>0.99	8/8 (100.0)	9/9 (100.0)	-
**Nasal cannula n (%)**	76/77 (98.7)	120/130 (92.3)	0.07	8/8 (100.0)	14/14 (100.0)	-	57/97 (58.8)	50/93 (53.8)	0.54	42/69 (60.9)	18/35 (51.4)	0.37	107/109 (98.2)	96/98 (98.0)	>0.99	7/8 (87.5)	9/9 (100.0)	>0.99
**Venturi mask n (%)**	6/77 (7.8)	17/130 (13.1)	0.23	0	1/14 (7.1)	>0.99	6/97 (6.2)	3/93 (3.2)	0.51	5/69 (7.2)	6/35 (17.1)	0.16	7/109 (6.4)	3/98 (3.1)	0. 36	4/8 (50.0)	2/9 (22.2)	0.32
**Reservoir mask n (%)**	8/77 (10.4)	22/130 (16.9)	0.21	2/8 (25.0)	6/14 (42.9)	0.64	3/97 (3.1)	2/93 (2.2)	>0.99	3/69 (4.3)	1/35 (2.9)	>0.99	0	0	-	0	0	-
**High-flow nasal cannula n (%)**	1/77 (1.3)	1/130 (0.8)	>0.99	7/8 (87.5)	7/14 (50.0)	0.15	46/97 (47.4)	46/97 (47.4)	0.87	56/69 (81.2)	23/35 (65.7)	0.11	3/109 (2.8)	0	0.25	6/8 (75.0)	7/9 (77.8)	>0.99
**Non-invasive ventilation (NIV) n (%)**	0	0	-	1/8 (12.5)	1/14 (7.1)	>0.99	0	0	-	15/69 (21.7)	5/35 (14.3)	. 0.44	0	0	-	0	0	-
**Invasive mechanical ventilation (IMV) n (%)**	0	0	-	4/8 (50.0)	6/14 (42.9)	>0.99	0	0	-	15/69 (21.7)	5/35 (14.3)	0.44	0	0	-	0	0	-

*Study sites as in Table 1. Data are not representative at the national level.

**”-“ indicates that the comparison was not calculable

4. Supplementary Table 3. Detailed societal cost (in 2025 PPD USD) per LRTI episode for RSV-positive and RSV-negative children <5 years by study sites *

	Argentina				Brazil				Colombia			
	RSV (+)		RSV (-)		RSV (+)		RSV (-)		RSV (+)		RSV (-)	
	Ward (n=77)	ICU (n=8)	Ward (n=130)	ICU (n=14)	Ward (n=97)	ICU (n=69)	Ward (n=93)	ICU (n=35)	Ward (n=109)	ICU (n=8)	Ward (n=98)	ICU (n=9)
Total Costs	3,479.22 (3,011.67-3,946.77)	22,099.02 (11,537.74-32,660.30)	3,249.91 (2,705.51-3,794.31)	12,252.92 (7,997.40-16,508.43)	959.15 (853.55-1,064.74)	3,769.76 (3,110.53-4,428.99)	885.20 (796.46-973.94)	3,639.20 (2,398.54-4,879.87)	2,593.32 (2,381.74-2,804.91)	6,002.29 (3,410.34-8,594.23)	2,346.77 (2,132.12-2,561.42)	7,933.06 (2,872.16-12,993.95)
Total Direct Costs	3,394.33 (2,947.05-3,841.62)	22,015.77 (11,274.79-32,756.76)	3,177.41 (2,643.88-3,710.93)	12,191.57 (7,995.71-16,387.42)	900.52 (794.46-1,006.57)	3,682.11 (3,023.25-4,340.97)	836.02 (754.14-917.89)	3,539.64 (2,341.52-4,737.77)	2,393.35 (2,195.22-2,591.49)	5,576.13 (3,271.05-7,881.21)	2,146.52 (1,943.11-2,349.93)	7,537.95 (2,432.48-12,643.42)
Total Direct Medical Costs	3,188.98 (2,784.60-3,593.36)	21,457.68 (10,946.91-31,968.45)	3,018.56 (2,504.77-3,532.35)	11,771.49 (7,721.52-15,821.47)	675.52 (592.81-758.22)	3,080.54 (2,489.38-3,671.69)	623.83 (561.27-686.38)	2,935.23 (1,853.89-4,016.57)	1,974.17 (1,802.94-2,145.39)	4,912.98 (2,800.98-7,024.98)	1,770.25 (1,598.35-1,942.14)	6,665.08 (1,827.34-11,502.83)
Bed-day cost	2,490.93 (2,148.97–2,836.50)	11,260.05 6,867.39 – 15,405.23	2,366.61 (1,963.65–2,865.52)	6,717.14 (4,313.11 – 9,262.58)	539.71 (470.42 – 612.04)	2,276.18 (1,920.74 – 2,645.29)	491.93 (443.75 – 538.84)	2,075.22 (1,509.25 – 2,762.46)	1,658.29 (1,507.41 – 1,813.17)	4101.07 (2,460.64 – 5,905.53)	1,479.10 (1,330.60 – 1,640.97)	4,957.73 (2,478.87 – 9,186.39)
Total Medications costs	142.62 (114.93 – 173.82)	5,461.62 (915.93 – 11,319.82)	150.00 (124.90 – 177.37)	1,804.83 (962.22 – 2,948.64)	28.2 (21.00 – 37.00)	150.36 (118.18 – 183.87)	31.64 (23.97 – 39.92)	166.50 (122.00 – 223.25)	39.26 (26.28 – 54.56)	135.43 (40.33 – 255.57)	24.51 (17.37 – 32.33)	168.47 (42.23 – 337.93)
Total antibiotics costs	14.49 (7.19–22.93)	2125.65** (149.34–5013.06)	14.65 (6.74–23.86)	719.62* (167.77-1464.56)	10.70 (5.20–17.98)	50.99 (37.42–66.21)	16.24 (9.79–23.50)	71.40 (43.18 – 108.14)	1.87(1.04 – 2.77)	19.73 (-)	5.46 (3.23–8.54)	46.19 (3.95 – 102.77)
Ceftriaxone	3.95 (.84-7.91)	59.88 (-)	4.35 (.83-8.71)	20.22 (-)	4.44 (.30-10.77)	20.57 (12.49-30.03)	1.77 (.13-4.02)	36.93 (16.48-64.82)	0		0	10.67 (-)
Meropenem	0	475.15 (-)	0	237.57 (-)	0		0	0.55 (-)	0		0	0
Ampicilin	7.92 (1.70 – 15.84)	0	7.71 (2.68–14.07)	0	0		0.25 (-)	0.44 (-)	0	0	0	0.63 (-)
Amoxycilin	1.32 (0.66 – 2.13)	0.77 (-)	0.83 (0.50–1.25)	0	3.48 (: 1.25 – 6.13)	0.28 (-)	5.81 (: 2.62 – 9.74)	6.95 (-)	1.31 (0.68–2.02)	0	1.48 (0.85-2.26)	0
Amxycilin-clavulanic	0	0	0.36 (-)	0.51 (-)	1.79 (0.45 – 3.58)	0.56 (-)	7.46 (3.03 – 13.06)	22.31 (11.78 – 34.09)	0	0	0	0
Claritromicin	0.51 (-)	2.99 (-)	0.47 (0.13 – 0.84)	4.36 (1.71 – 7.78)	0	5.88 (-)	0	0	0	0	0.80 (-)	0

Ampicilin-sulbactam	0	178.18 (-)	0	188.36 (-)	0	18.55 (9.43 – 28.92)	0	0	0.34 (-)	12.30 (-)	0.16 (-)	1.69 (0; 4.58)
Cefotaxime	0	0	0	0	.10 (-)	2.54 (-)	0	0	0	0	0	0
Clindamicin	0	0	0.14 (-)	0	0.23 (-)	0	0	1.40 (-)	0	4.34 (1.09; 8.68)	1.14 (-)	28.01 (-)
Piperaacilin-tazobactam	0	1199.00 (-)	0	252.42 (-)	0	0	0	0	0	0	0	0
Gentamicin	0	0	0	0	0	0.14 (-)	0	0.15 (-)	0	0	0.02 (-)	0.51 (-)
Amikacin	0	0	0	0	0.26 (-)	0	0	0	0	0	0	0
Penicilin	0	0	0	0	0.39 (-)	0	0.95 (0.27-1.90)	0	0		0.35 (0.07 – 0.71)	0
Vancomicin	0	207.87 (-)	0	0	0	0.21 (-)	0	2.38 (-)	0	0	0	1.92 (-)
Oseltamivir	0.78 (0.46 – 1.19)	1.78 (-)	0.79 (0.40 – 1.18)	3.73 (1.47 – 6.22)	0	0.18 (-)	0	0	0.23(-)	0	1.51 0.50 – 2.77	2.74 (-)
Total oral corticosteroids	47.43 (27.93 – 68.64)	269.50 (110.25 – 457.33)	49.72 (36.41 – 64.98)	269.04 (182.46– 360.53)	0.03 (-)	2.08 (-)	0.16 (-)	0.90 (0.16 – 1.88)	0.07 (0.03 – 0.11)	0	0.05 (0.01 – 0.10)	0.73 (0.30 – 1.23)
Methylprednisone	23.75 (10.08 – 39.96)	0	27.72 (17.48 – 39.66)	19.80 (-)	0.03 (-)	0	0.12 (-)	0.32 (-)	0.04 (0.01 – 0.07)	3.09 (-)	0.04 (0.00 – 0.10)	0.51 (-)
Betametasone	0.38(-)	0	0.13 (-)	0	0	2.29 (0.37 – 5.79)	0	0	0	0.78(-)	0	0
Hydrocortisone	8.02 (2.67– 14.71)	0	5.54 (1.98– 9.50)	88.24 (29.41 – 183.84)	0	0.28 (-)	0	0.22 (-)	0	0.72 (-)	0	0
Dexametasone	15.27 (6.36 – 25.24)	269.50 (110.25 – 457.33)	16.33 (9.93– 23.87)	161.00 (86.33 – 234.50)	0	1.60 (-)	0.04 (-)	0.35 (-)	0.03 (0.01 – 0.06)	0	0.01 (-)	0.22 (-)
Total bronchodilators	74.63 (62.15 – 85.97)	270.61 (150.46 – 406.23)	81.86 (66.79 – 100.36)	261.76 (169.38-357.39)	10.54 (8.02 – 13.15)	23.79 (16.37-31.20)	10.97 (8.82 – 13.55)	11.65 (6.04 – 17.82)	32.22 (20.52 – 46.59)	189.16 (37.15-341.16)	14.71 (8.52 – 21.16)	104.07 (3.28 – 277.25)
Albuterol	63.15 (53.17 – 72.40)	174.22 (95.03 – 257.37)	59.70 (48.92 – 72.43)	175.35 (114.83 – 253.41)	9.70 (7.49– 11.97)	0.41 (0.16– 0.72)	9.94 (8.05– 12.20)	8.54 (4.96 – 12.23)	8.71 (7.50 – 9.96)	0.06 (-)	5.71 (4.60 – 6.84)	6.77 (1.44 – 13.64)
Ipratropium	11.48 (6.72–	96.39 (30.56–	19.10 (13.17–	64.48 (25.53–	0.84 (0.28–	11.90 (8.53 –	1.02 (0.29 –	3.11 (-)	6.14 (2.89 –	94.58 (28.16 –	1.20 (-)	32.78 (-)

	16.61)	188.08)	25.25)	107.47)	1.54)	15.44)	1.83)		10.11)	173.56)		
Budesonide	0	0	3.07 (-)	21.92 (-)	0	7.72 (5.38–10.01)	2.59 (0.32–4.87)	0	17.38 (5.82–28.94)	12.69 (8.31 – 16.15)	7.80 (2.24–13.35)	64.52 (-)
Total analgesics	5.16 (1.80–9.48)	111.44** (18.56–256.18)	3.46 (1.29–6.11)	19.09 (-)	1.85 (1.35–2.38)	1.58 (0.39–3.15)	1.35 (0.94–1.80)	5.58 (3.19–8.80)	4.67 (3.29 – 6.16)	24.59 (-)	3.72 (2.55–4.78)	13.91 (-)
Acetaminophen	0.02 (-)	0.08 (-)	0.02 (-)	0	1.10 (0.61 – 1.62)	0	0.72 (0.34 – 1.18)	3.04 (1.57 – 4.72)	4.21 (3.00 – 5.48)	57.30 (-)	3.34 (2.34–4.30)	13.65(-)
Ibuprofen	3.21 (0.80 – 6.02)	0	0.48 (-)	0	0	4.96 (3.54–6.62)	0	0	0.43 (-)	10.97 (4.50–17.11)	0.29 (-)	0
Dypirone	1.93 (-)	111.36 (18.56 – 256.14)	2.97 (0.91 – 5.48)	19.09 (-)		3.19 (1.97 – 4.59)			0.03 (-)	10.75 (4-.09–16.89)	0.09 (0.03 – 0.17)	0.26 (-)
Total vasoactive drugs	0	23.02 (-)	0	5.30 (-)	0.02 (-)	0.08 (-)	0.02 (-)	3.34 (0.03 – 8.85)	0	0	0.01 (-)	0.14 (-)
Adrenaline	0	0	0	0	0.02 (-)		0.02 (-)	0.07 (0.023 – 0.127)	0	0.22 (-)	0.01 (-)	
Dopamine	0		0	0	0	0.18 (0.08 – 0.31)	0	0	0	0.05 (-)	0	0
Milrinone	0	15.59 (-)	0	0	0	0.15 (0.076 – 0.240)	0	3.19 (-)	0		0	0
Dobutamine	0		0	0	0	0	0	0	0		0	0
Noradrenaline	0	7.42 (-)	0	5.30(-)	0		0	0.08 (-)	0	0.05 (-)	0	0
Any sedation-analgesia	0	775.58 (229.41–1318.04)	0	440.93 (153.01–766.04)	0	0	0	21.20 (7.91 – 42.59)	0	0	0	0
Midazolam	0	147.00 (-)	0	40.73 (-)	0.02 (-)	0.03 (-)	0	1.72	0	0	0	0

								(0.47 – 3.75)				
Fentanyl	0	420.71 (123.74 – 717.67)	0	212.12 (53.03 – 395.96)	0	0	0	4.46 (0.85 – 9.55)	0	0	0	0
Ketamine	0	207.88 (-)	0	188.08 (-)	0	21.66 (12.41–32.22)	0	15.02 (5.90 – 30.04)	0	0	0	0
Total diuretics	0	161.11 (-)	0	82.44 (16.21 – 160.95)	0.01(-)	0.69 (0.35 – 1.04)	0	0.26 (-)	0.03 (-)	6.77 (-)	0	0.31 (-)
Furosemide	0	161.11 (-)	0	80.18 (-)	0.01 (-)	7.65 (4.31 – 11.47)	0	0.26 (-)	0.03 (-)	0	0	0.31 (-)
Spironolactone	0	0	0	2.26 (-)	0	13.33 (7.07–20.27)	0	0	0	0	0	0
Enteral nutrition	0.30 (-)	0.67 (-)	0.04 (-)	2.04 (-)	4.81 (2.33 – 7.76)	2.55 (0.61–4.95)	2.75 (0.73 – 5.42)	51.16 (31.17 – 77.60)	0	6.77 (-)	0	0
Hydration IV	0.55 (0.19–1.00)	4.56 (2.22–7.13)	0.21 (0.05 – 0.42)	2.47 (-)	0.06 (-)	0.44 (0.17–0.76)	0.08 (-)	0.97 (0.47–1.67)	0.31 (0.07 – 0.60)	0	0.52 (0.22–0.93)	3.07 (-)
Parenteral Nutrition	0	1715.46 (-)	0	0	0	2.11 (0.37–4.22)	0	0	0	0	0	0
Total imaging	26.61 (20.44-34.32)	123.49 (69.66-181.15)	25.55 (20.07-32.40)	68.44 (44.33-94.53)	5.76 (3.42-9.81)	54.74 (43.94–65.10)	4.21 (3.49-4.96)	29.76 (15.14-49.80)	114.68 (91.73-140.16)	222.78 (89.62-355.93)	117.25 (92.32-145.98)	535.64 (267.27-860.90)
Radiography	21.98 (18.13–25.45)	55.68 (40.83–70.53)	20.79 (17.36–23.99)	48.79 (36.06–63.64)	3.94 (3.11–4.77)	1.03 (0.65–1.42)	3.81 (3.24–4.32)	13.10 (9.59 - 17.70)	68.11 (61.09 – 75.13)	124.37(76.54 – 172.21)	72.63 (65.60–79.66)	314.65(161.58–484.73)
Ultrasound	0	21.53 (-)	3.09 (0.88–6.18)	4.10 (-)	0.32 (-)	0	0.33 (-)	1.76 (-)	0	0	0	64.69 (-)
Computed Tomography (CT)	0	0	0.76 (-)	7.07 (-)	1.19 (-)	21.19 (14.34-29.31)	0	13.20 (3.30-26.40)	2.84 (-)	0	0	68.83 (-)
Electrocardiogram (ECG)	0	1.73 (-)	0	0	0.02 (-)	12.36 (10.05 – 14.86)	0.07 (-)	0.06 (-)	0.39 (-)	0	0.44 (-)	0
Echocardiogr	4.63 (-)	44.55 (-)	0.91 (-)	8.48 (-)	0.30 (-)	0.45 (-)	0	1.64 (-)	43.33	98.41 (-)	44.18	87.47 (-)

aphy									(21.67-68.61)		(24.10-68.28)	
Total laboratory tests	0.94 (0.19–2.00)	28.81 14.01 – 46.25	1.37 (0.63–2.41)	22.24 (7.54–42.68)	2.18 (1.03–3.63)	6.70 (1.67.11.72)	0.85 (0.45–1.31)	22.30 (14.75 – 32.22)	19.77 (12.69 – 27.27)	71.30 (34.45–107.82)	18.85 (12.85 – 26.30)	430.91 (137.38 – 831.06)
Complete Blood Count (CBC) n (%)	0.16 (0.05–0.32)	3.56 (2.00–5.35)	0.30 (0.18–0.44)	2.93 (1.15–5.47)	0.52 (0.29–0.81)	0.03 (-)	0.30 (0.17–0.45)	3.23 (2.39–4.40)	5.06 (3.46–6.66)	16.34 (10.89–21.78)	5.04 3.70 – 6.67	58.08 (19.36 - 107.29)
Coagulation profile n (%)	0	3.12 (-)	0.10 (-)	2.38 (0.59–4.45)	0.15 (-)	1.67 (-)	0.05 (-)	1.37 (0.55–2.61)	0.25 (-)	0	0.28 (-)	6.01 (-)
Hepatic profile n (%)	0	0	0	1.91 (0.76–3.31)	0.18 (0.04 – 0.39)	19.80 (14.05–26.97)	0.09 (0.02 – 0.16)	0.63 (0.29 – 1.07)	0.56 (-)	0	0.62 (-)	16.91 (-)
Renal function n (%)	0.12 (-)	4.16 (1.78–6.53)	0.31 (0.16 – 0.48)	2.55 (0.85 – 4.58)	0.21 (0.10)–0.36)	2.80 (2.12–3.64)	0.05	2.55 (1.75 – 3.62)	0.99 (0.28 – 1.70)	5.77 (-)	0.79 (0.16–1.57)	18.82 (-)
Erythrocyte Sedimentation Rate n (%)	0	0	0.02 (-)	0	0	0.77 (0.35–1.25)	0	0	0.03 (-)	0	0	0
C-reactive protein (PCR) n (%)	0.42 (-)	5.20 (2.60 – 7.80)	0.21 (0.07 – 0.37)	2.55 (1.06–4.24)	0.30 (0.17–0.43)	0.30 (0.15 – 0.48)	0.17 (0.09–0.24)	2.12 (1.54 – 2.84)	7.31 (4.60 – 10.29)	25.83 (11.07 – 40.59)	7.83 (5.12–11.15)	72.16 (29.52 – 131.20)
Blood gases n (%)	0.05(-)	7.28 (-)	0.19 (-)	8.32 (-)	0.75 (0.27 – 1.37)	2.29 (1.54-3.21)	0.14 (-)	9.84 (5.68 – 15.05)	1.22 (-)	0	0	176.84 (-)
Ionogram n (%)	0.10 (-)	2.77 (0.99-5.35)	0.12 (0.05 – 0.21)	2.26 (0.40–5.32)	0.15 (0.06–0.24)	0.02(-)	0.05 (-)	2.69 (1.70–3.87)	4.49 (2.04 – 7.15))	22.27 (-)	4.09 (1.82–6.82)	94.03 (22.27 – 188.05)
Glucose n (%)	0.05 (-)	2.23 (0.67–3.79)	0.12 (0.04–0.22)	1.27 (0.38 – 2.29)	0	1.86 (1.35 – 2.47)	0	0	0.16 (-)	1.09 (-)	0.45 (-)	2.92 (-)
Urinalysis n (%)	0.10 (-)	2.77 (0.99-5.35)	0.12 (0.05-0.21)	2.26 (0.40-5.32)	0.15 (0.06–0.24)	9.31 (6.24–13.25)	0.05 (-)	2.69 (1.70 – 3.87)	4.49 (2.04 – 7.15)	22.27 (-)	4.09 (1.82–6.82)	94.03 (22.27 – 188.05)
Total Microbiological Culture	16.02 (7.46 – 26.26)	153.93 (66.69 – 290.41)	7.70 (3.09 – 13.13)	83.86 (41.33 – 133.71)	1.04 0.51 – 1.70	2.16 (1.44–3.09)	0.74 (0.37 – 1.17)	7.96 (5.42 – 10.98)	2.64 (-)	25.48 (-)	3.35 (-)	53.01 (-)
Hemoculture	15.66 (7.46–	129.18 (57.41–	7.51 (3.09–12.81)	69.72 (32.81 –	0.72 (0.29–	0.01	0.60 (0.22–	4.78 (3.39–	2.28 (-)	20.74 (-)	2.96 (-)	36.88 (-)

	26.10)	244.01)		106.63)	1.22)		1.05)	6.37)				
Urine Culture	0	15.47 (-)	0.19 (-)	5.30 (-)	0.27 (0.09-0.50)	2.16 (1.44–3.09)	0.14 (-)	1.27 (0.38–2.29)	0.35 (-)	0	0.39 (-)	8.51 (-)
Cerebrospinal Fluid (CSF) Culture n (%)	0	0	0	0	0	8.48 (6.33 – 10.86)	0	0.13(-)	0	0	0	0
Pleural Fluid Culture	0	0	0	1.77 (-)	0.05 (-)	5.25 (4.04–6.57)	0	0.51 (-)	0	0	0	7.62 (-)
Tracheal Aspirate Culture n (%)	0	9.28 (-)	0	7.07 (-)	0	1.74 (1.16–2.45)	0	0.76 (0.19–1.52)	0	4.74 (-)	0	0
Stool Culture n (%)	0.36 (-)	0	0	0	0	0.06 (-)	0	0.51 (-)	0	0	0	0
Respiratory Virus Test*	293.11 (285.40–296.97)	296.97 (296.97–296.97)	299.25 (280.98 – 317.53)	403.03 (318.18 – 509.09)	37.97 (36.01–39.93)	0.06 (-)	38.46 (35.96 – 40.97)	44.15 (40.52 – 48.99)	92.53 (88.47–97.40)	121.65 (88.47–154.83)	90.28 (88.47–92.99)	147.46 (117.96–176.95)
Peritoneal dialysis n (%)	0	0	0	0	0	1.16 (0.64-1.80)	0	1.35 (-)	0	0	0	0
Total oxygen requirement	218.81 (152.9–322.6)	4132.81 (2495.8–6063.7)	168.13 (140.3–199.9)	2672.01 (1792.4 – 3578.5)	60.62 (50.1–71.6)	563.48 (340.66-786.29)	56.07 (46.3–65.5)	588.06 (167.9–1195.7)	47.10 (41.0 – 54.1)	235.35 (163.12-307.57)	36.95 (32.7–41.6)	371.91 (145.3 – 763.9)
Nasal cannula	158.38 (135.24 – 186.67)	74.24 (59.39–89.09	127.01 (105.99 – 152.60)	101.82 (65.05 – 148.48)	10.81 (7.83–14.24)	41.11 (38.96–43.25)	9.96 (7.38–13.17)	11.67 (6.32–18.95)	40.60 (36.54–44.96)	36.21 (22.13–48.28)	36.13 (31.98–40.76)	67.95 (36.65–102.81)
Venturi mask	8.36 (1.93–16.71)	0	14.47 (7.61–22.46)	14.14 (7.94–21.62)	1.63 (0.33–3.80)	1.22 (.15-2.28)	0.91 (-)	2.41 (0.60 – 4.51)	2.36 (0.74 – 4.58)	22.13 (-)	0.82 (-)	3.58 (-)
Reservoir mask	7.07 (2.57–12.86)	12.37 (-)	15.99 (8.76–24.37)	31.82 (14.14 – 53.03)	0.33 (-)	0.46 (-)	0.34 (-)	0.30	0	0	0	0
High-flow nasal cannula	45.00 (-)	1,905.55 (1,039.39 – 2,944.94)	10.66 (-)	1,385.85 (593.94 – 2,227.26)	46.78 (35.62–59.28)	87.50 (70.49-104.50)	43.19 (32.39–53.70)	64.08 (41.73–87.93)	4.13 (-)	177.02 (98.06-255.96)	0	200.26 (71.52 – 393.37)
Non-invasive ventilation (NIV)	0	61.87 (-)	0	35.35 (-)	1.08 (-)	19.65 (12.55 - 26.75)	1.68 (-)	17.88 (8.57–27.57)	0	0	0	100.13 (-)

Invasive mechanical ventilation (IMV)	0	2,078.78 (-)	0	1,103.03 (367.68 – 1,909.08)	0	444.09 (217.47-670.70)	0	491.72 (71.96–1,109.37)	0	0	0	0
Total Direct non-Medical Costs	205.35 (141.04-269.65)	558.09 (321.41-794.76)	158.84 (131.95 185.73)	420.07 (249.56-590.58)	225.00 (200.36-249.63)	601.57 (523.83-679.30)	212.19 (190.72-233.65)	604.40 (460.30-748.51)	419.18 (382.22-456.15)	663.15 (414.39-911.91)	376.27 (336.34-416.20)	872.86 (516.00-1229.72)
Operational Overhead***	91.75 (79.48-104.03)	487.64 (298.64-676.40)	87.17 (69.96-104-40)	374.92 (247.77-502.07)	168.31 (147.11-189.41)	488.05(218.56-343.87)	153.42 (137.78-168.94)	526.24 (394.97-657.50)	221.56 (201.81-241.32)	442.37 (323.62 - 561.12)	197.62 (177.45-217.79)	531.50 (264.90-798.08)
Transportation	54.20 (25.01-83.39)	60.59	34.84 (23.70-45.98)	34.22 (-)	47.30 (39.40-55.21)	91.12 (64.20-118.05)	46.49 (62.92–82.94)	66.42 (41.86–104.01)	120.82 (102.70-138.93)	142.64 (52.89-232.39)	114.38 (96.29-132.47)	196.63 (134.39-258-87)
Accommodation	0	0	0	0	0	0	0	0	0	(41.73–87.93)	0.68	78.02 (-)
Paid Caregiver	29.20 (0 – 75.00)	0	12.86 (4.98-20.74)	9.90 (-)	5.44 (1.16-9.71)	19.42 (7.49-31.34)	8.85 (4.04-13.65)	8.78 (1.39-16.16)	45.85 (28.53-63-15)	0	37.01 (21.11-52-90)	42.13 (-)
Meals	30.18 19.47-40.90	9.86 (-)	23.96 (17.16-30.75)	1.03 (-)	3.99 (2.56-5.43)	2.97 (1.63-4.30)	3.48 (2.27-4.70)	2.98 (1.77-4.18)	30.95 (22.34-39.56)	0	26.57 (21.49-31.64)	24.57 (14.59-34.56)
Indirect costs (productivity losses)	84.89 (61.07-108.70)	83.25 (-)	72.51 (51.45-93.56)	61.35 (-)	58.63 (43.45–73.81)	87.65 (-)	49.19 (33.98–64.39)	99.56 (57.60–141.52)	199.97 (158.93–241.01)	426.16 (267,03-588,84)	200.25 (158.43–242.06)	395.11 (151.88–638.34)

**Given the highly right-skewed distribution of cost data and the presence of a large proportion of zero values, percentile-based bootstrap confidence intervals were estimated for mean costs. This approach avoids reliance on normality assumptions and prevents the generation of implausible negative confidence limits, providing more robust and interpretable uncertainty estimates for average per-patient costs. Bootstrap confidence intervals were not estimated for country–severity strata with fewer than five observations with non-zero costs, as resampling in very small effective samples leads to unstable and non-informative interval estimates.*

**In Argentina ICU patients, antibiotic costs were highly right-skewed; the median [IQR] cost was USD 244 [109–2,330], substantially lower than the mean. Among non-ICU Argentine patients, median [IQR] antibiotic costs were USD 226 [0–516] in RSV-positive cases and USD 0 [0–8] in RSV-negative cases, reflecting infrequent use with occasional high-cost outliers.

*** Includes utilities and support services allocated by patient-days (electricity, water, gas, internet, taxes)*Study sites as in Table 1. Data are not representative at the national level.

4. Supplementary Table 4. *Societal cost (in 2025 OER USD) per LRTI episode for RSV-positive and RSV-negative children <5 yearsby study sites**

	Argentina				Brazil				Colombia			
	RSV (+)		RSV (-)		RSV (+)		RSV (-)		RSV (+)		RSV (-)	
	Ward (n=77)	ICU (n=8)	Ward (n=130)	ICU (n=14)	Ward (n=97)	ICU (n=69)	Ward (n=93)	ICU (n=35)	Ward (n=109)	ICU (n=8)	Ward (n=98)	ICU (n=9)
**Total Costs**	1,523.39 (1,318.67-1,728.11)	9,676.14 (5,051.85-14,300.44)	1,422.99 (1,184.62-1,661.35)	5,364.99 (3,501.69-7,228.28)	417.52 (371.55-463.48)	1,640.98 (1,354.02-1,927.94))	385.33 (346.70-423.96)	1,584.15 (1,044.09-2,124.21)	923.69 (848.33-999.06)	2,137.91 (1,214.70-3,061.11)	835.88 (759.42-912.33)	2,825.61 (1,023.01-4,628.21)
**Direct Costs**	1,486.22 (1,290.38-1,682.07)	9,639.69 (4,936.71-14,342.68)	1,391.24 (1,157.63-1,624.85)	5,338.13 (3,500.95-7,175.30))	392.00 (345.83-438.16)	1,602.83 (1,316.02-1,889.63)	363.92 (328.28-399.56)	1,540.81 (1,019.27-2,062.36)	852.47 (781.90-923.04)	1,986.12 (1,165.09-2,807.15)	764.55 (692.10-837.00)	2,684.88 (866.41-4,503.36)
**Direct Medical Costs**	1,396.31 (1,219.25-1,573.37)	9,395.33 (4,793.15-13,997.51)	1,321.69 (1,096.72-1,546.65)	5,154.19 (3,380.90-6,927.49)	294.05 (258.05-330.05)	1,340.96 (1,083.63-1,598.29)	271.55 (244.32-298.78)	1,277.71 (807.00-1,748.42)	703.16 (642.17-764.15)	1,749.92 (997.66-2,502.17)	630.53 (569.30-691.76)	2,373.98 (650.87-4,097.10)
**Total Direct non-Medical Costs**	89.91 (61.75-118.07)	244.36 (140.73-347.99)	69.55(57.17–81.24)	183.93 (109.27-258.59)	97.94 (87.22-108.66)	261.86 (228.02-295.70)	92.37 (83.02-101.71)	263.10 (200.37-325.83)	149.30 (136.14-162.47)	236.20 (147.60-324.81)	134.02 (119.80-148.24)	310.90 (183.79-438.00)
**Total Indirect costs (productivity losses)**	37.17 (26.74-47.59)	36.45 (-)	31.75 (22.53-40.97)	26.86 (-)	25.52 (18.91-32.13)	38.15 (-)	21.41 (14.79-28.03)	43.34 (25.07-61.60)	71.23 (56.61-85.84)	151.79 (95.11-209.73)	71.33 (56.43-86.22)	140.73 (54.10-227.37)

*Study sites as in Table 1. Data are not representative at the national level.

5. Supplementary Table 5. DALYs per episode by study site* and level of care

Country	Care	n	Median DALY per episode*	IQR (P25–P75)
Argentina	RSV + Ward	77	0,0011	0,0007-0,0022
Argentina	RSV + ICU	8	0,0058	0,0029-0,0075
Argentina	RSV - Ward	130	0,0011	0,0007-0,0018
Argentina	RSV – ICU	14	0,0052	0,0023-0,0063
Brazil	RSV + Ward	97	0,0011	0,0007-0,0015
Brazil	RSV + ICU	69	0,0035	0,0023-0,0052
Brazil	RSV - Ward	93	0,0011	0,0007-0,0018
Brazil	RSV – ICU	35	0,0032	0,0023-0,0046
Colombia	RSV + Ward	109	0,0018	0,0011-0,0026
Colombia	RSV + ICU	8	0,0052	0,0037-0,0055
Colombia	RSV - Ward	98	0,0015	0,0011-0,0022
Colombia	RSV - ICU	9	0,0075	0,0029-0,0104

*DALYs showed a highly right-skewed distribution driven by rare fatal events. In small subgroups, mean estimates and bootstrap uncertainty intervals were unstable. Therefore, DALYs are primarily reported as medians with interquartile ranges.

*Study sites as in Table 1. Data are not representative at the national level.

6. Supplementary Figure 1.

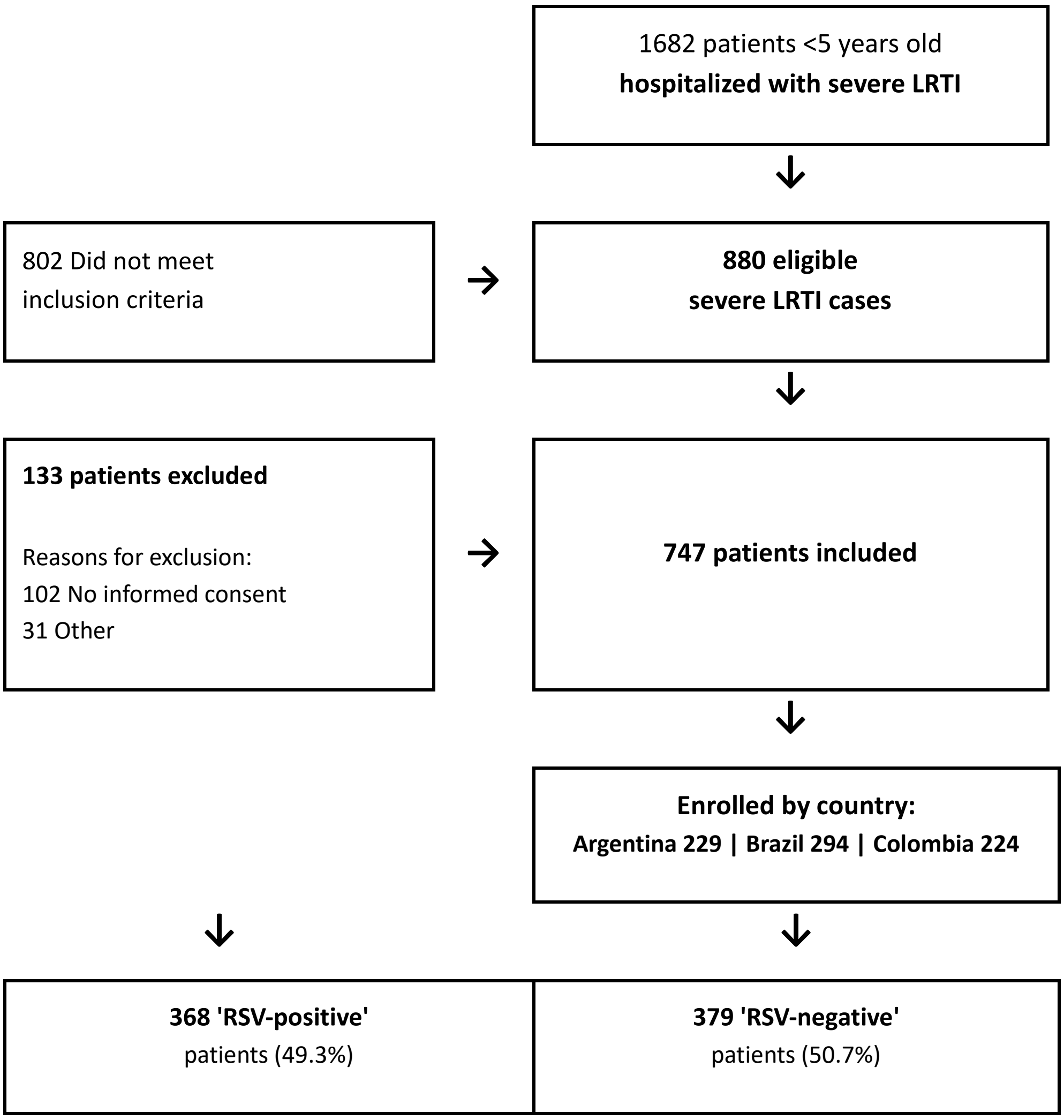

7. Supplementary Figure 2.

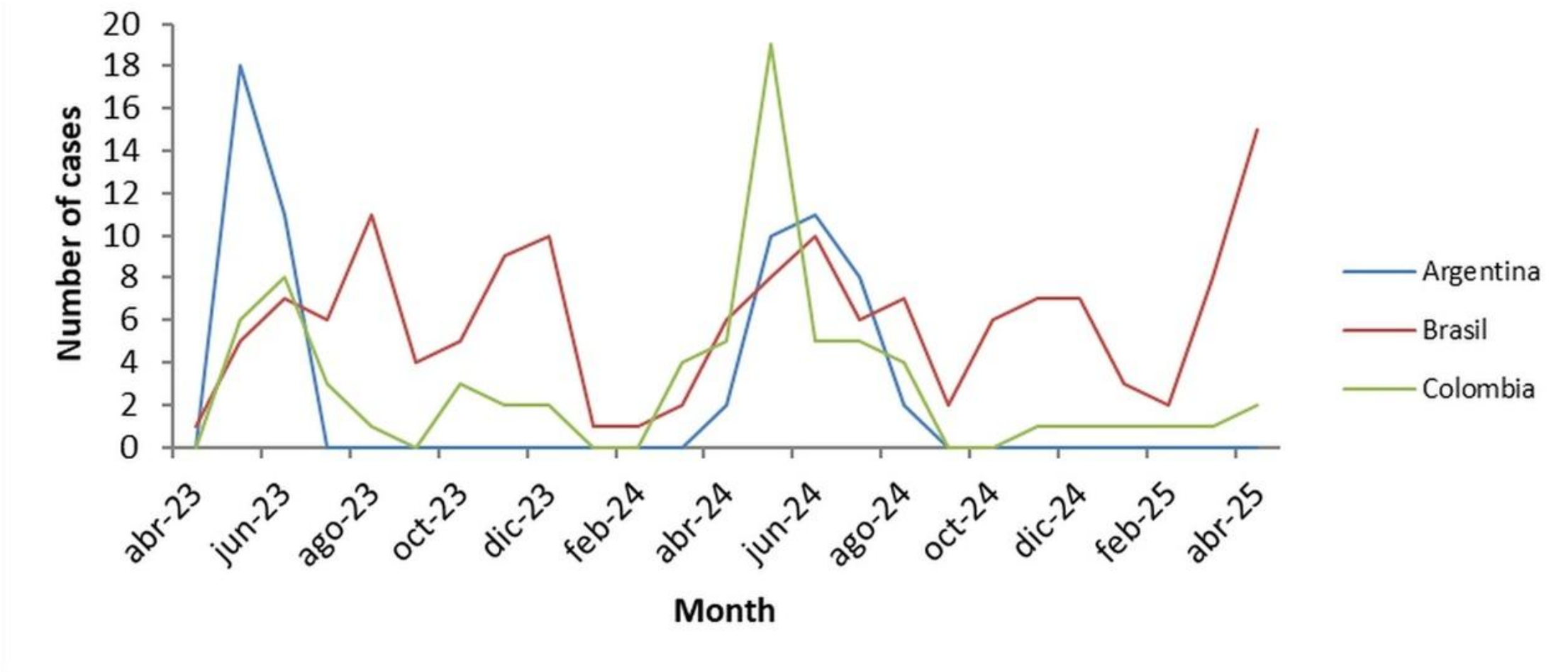

8. References

1. Onwuchekwa C, Atwell J, Moreo LM, *et al.* Pediatric Respiratory Syncytial Virus Diagnostic Testing Performance: A Systematic Review and Meta-analysis. *The Journal of Infectious Diseases* 2023; **228**: 1516–27.

2. Kuypers J, Wright N, Morrow R. Evaluation of quantitative and type-specific real-time RT-PCR assays for detection of respiratory syncytial virus in respiratory specimens from children. *Journal of Clinical Virology* 2004; **31**: 123–9.

3. Drummond MF, Sculpher MJ, Claxton K, Stoddart GL, Torrance GW. Methods for the Economic Evaluation of Health Care Programmes. 4th ed. Oxford University Press; 2015.

4. Tan SS, Rutten FFH, van Ineveld BM, Redekop WK, Hakkaart-van Roijen L. Comparing methodologies for the cost estimation of hospital services. Eur J Health Econ. 2009;10(1):39-45.
5. Oostenbrink JB, Rutten FFH. Cost assessment and price setting of inpatient care in the Netherlands. Health Care Manag Sci. 2006;9(3):287-296.
6. Kahn JM, Rubenfeld GD. Resource intensity of intensive care units. Curr Opin Crit Care. 2010;16(6):593-598.
7. Halpern NA, Pastores SM. Critical care medicine in the United States 2000–2005. Crit Care Med. 2010;38(1):65-71.
8. Gold MR, Siegel JE, Russell LB, Weinstein MC. Cost-Effectiveness in Health and Medicine. Oxford University Press; 1996.
9. Sculpher MJ, Pang FS, Manca A, et al. Generalisability in economic evaluation studies in healthcare. Health Technol Assess. 2004;8(49):1-192.
10. Hutubessy R, Chisholm D, Edejer TT-T. Generalized cost-effectiveness analysis for national-level priority-setting in the health sector. Cost Eff Resour Alloc. 2003;1(1):8.
11. World Health Organization. WHO Guide to Cost-Effectiveness Analysis. WHO; 2003.
12. Jo C. Cost-of-illness studies: concepts, scopes, and methods. Clin Mol Hepatol. 2014;20(4):327-337.
13. Efron B, Tibshirani RJ. An Introduction to the Bootstrap. Chapman & Hall; 1993.
14. Briggs AH, Wonderling DE, Mooney CZ. Pulling cost-effectiveness analysis up by its bootstraps. Health Econ. 1997;6(4):327-340.

9. Figure legends

Supplementary Figure 1. Study participant flow diagram. LRTI, lower respiratory tract infection; RSV, respiratory syncytial virus.

Supplementary Figure 2. Monthly circulation of RSV-positive pediatric cases, May 2023–May 2025. Number of RSV-positive cases per month by study site. RSV, respiratory syncytial virus.
